# Coronary artery calcium burden and metabolic indices in relation to arteriovenous fistula functionality and post-functionality failure

**DOI:** 10.3389/fsurg.2026.1916736

**Published:** 2026-09-28

**Authors:** Kelong Shi, Jiang Xu, Dongmei Duan, Mingxu Ou, Guchao Zhou

**Affiliations:** 1 Tongling People’s Hospital, Tongling, Anhui, China; 2 Bengbu Medical University, Bengbu, Anhui, China

**Keywords:** arteriovenous fistula, vascular calcification, renal dialysis, chronic kidney failure, competing risk

## Abstract

**Background:**

Autogenous arteriovenous fistula (AVF) is the preferred haemodialysis access, but functionality failure and subsequent post-functionality AVF failure remain common. Whether coronary artery calcium (CAC), a marker of systemic vascular calcification, is associated with either outcome is unclear; competing events are frequently ignored.

**Objective:**

To examine CAC and metabolic markers in relation to AVF functionality and post-functionality AVF failure.

**Methods:**

In this single-centre retrospective cohort, 645 patients with end-stage kidney disease underwent first-time radiocephalic AVF creation. The primary outcome was functionality failure (*n* = 139 events; assisted functionality or abandonment before cannulation), with unassisted functionality, death and transplantation as competing events. A secondary outcome was post-functionality AVF failure among 568 fistulas that achieved successful functionality, with death and transplantation as competing events. Fine–Gray subdistribution hazard models were fitted after multiple imputation (*n* = 20).

**Results:**

Independent predictors of functionality failure were age (subdistribution hazard ratio [SHR] 1.53, 95% confidence interval [CI] 1.22–1.93, *P* < 0.001 per 1 standard deviation [SD]), CAC score (SHR 1.21, 95% CI 1.06–1.38, *P* = 0.004 per 1 SD) and left ventricular diastolic dysfunction (SHR 1.44, 95% CI 1.02–2.04, *P* = 0.040). Categorically, CAC 1–100 and >100 vs. 0 yielded SHRs of 5.38 (95% CI 3.02–9.58) and 6.61 (95% CI 3.57–12.22), respectively, both *P* < 0.001. For post-functionality AVF failure, assisted functionality (SHR 1.68, 95% CI 1.25–2.25, *P* < 0.001), diabetes (SHR 1.42, 95% CI 1.07–1.89, *P* = 0.015), serum albumin (SHR 0.71, 95% CI 0.56–0.88, *P* = 0.002 per 1 SD) and C-reactive protein (SHR 0.78, 95% CI 0.64–0.97, *P* = 0.022 per 1 SD) were independently associated. The C-index was 0.746 (95% CI 0.70–0.79) at 180 days for the primary outcome and 0.677 (95% CI 0.632–0.725) at 2 years for the secondary outcome.

**Conclusions:**

Older age and higher CAC burden are independently associated with AVF functionality failure, whereas the strongest determinant of subsequent patency loss is the need for assisted functionality. Preoperative static markers discriminate moderately; postoperative haemodynamic parameters are likely required for individual risk prediction.

## 1 Introduction

Autogenous arteriovenous fistula (AVF) remains the recommended vascular access for maintenance haemodialysis because of its low infection rate, reduced all-cause mortality, and superior long-term patency compared with central venous catheters and synthetic grafts. International guidelines consistently designate AVF as the first-choice access whenever anatomically feasible, reflecting its pivotal role as the “lifeline” for patients with end-stage kidney disease (ESKD) (1). However, the clinical utility of AVF is substantially undermined by two sequential hurdles that impose enormous physical, psychological, and economic burdens on patients and healthcare systems alike. First, poor functionality occurs in 20%–50% of newly created fistulas, necessitating repeated radiological or surgical interventions, prolonged catheter dependence with attendant infection and central venous stenosis risks, and markedly increased healthcare expenditure (2). Second, even after successful functionality, 30%–40% of AVFs fail within the first year because of thrombosis, progressive stenosis, or inadequate flow, exposing patients to recurrent hospitalisation, temporary catheter reinsertion, and access-related morbidity (3). Accurate identification of preoperative risk factors—both modifiable and non-modifiable—is therefore essential for optimising surgical decision-making, counselling patients realistically, and individualising postoperative surveillance intensity.

Coronary artery calcium (CAC), traditionally quantified by the Agatston score on non-contrast computed tomography, is a robust surrogate marker of systemic atherosclerotic burden and vascular calcification (4). Beyond the coronary tree, CAC reflects diffuse mineral deposition across the entire arterial bed, including the radial artery and cephalic vein that constitute the AVF circuit. In patients with ESKD, deranged calcium–phosphorus metabolism, secondary hyperparathyroidism, chronic inflammation, and uraemic toxins accelerate medial and intimal calcification, rendering vascular beds rigid and biologically senescent (5). Recent advances in artificial intelligence have enabled automated, reproducible CAC scoring from routine non-contrast chest CT without electrocardiogram gating, markedly enhancing clinical accessibility and making CAC a potentially scalable preoperative biomarker (6, 7). Despite these developments, empirical evidence linking preoperative CAC to AVF outcomes remains scarce.

Methodologically, prior investigations suffer from two interrelated limitations. First, the majority employ conventional Cox proportional hazards models that treat competing events—most notably death and kidney transplantation—as simple censoring mechanisms (8–10). This analytical strategy introduces informative censoring bias: patients who die or receive a transplant before experiencing the primary endpoint are wrongly assumed to have the same future risk as those remaining under follow-up, leading to overestimation of failure probabilities and distorted hazard ratios. In ESKD populations, where annual mortality exceeds 10% and transplantation rates continue to rise, such bias is particularly problematic and can fundamentally alter clinical conclusions. Second, functionality and patency loss have almost invariably been studied as isolated phenomena, without a unified two-stage analytical framework that mirrors the actual clinical trajectory of AVF from creation through functionality to long-term maintenance (11, 12). functionality failure is driven primarily by early postoperative vascular remodelling failure, whereas post-functionality AVF failure reflects chronic intimal hyperplasia, thrombosis, and haemodynamic deterioration; the biological drivers of these two stages are likely fundamentally different (11). Consequently, whether CAC exerts differential or shared effects across these consecutive stages remains entirely unknown, precluding a comprehensive understanding of how systemic vascular calcification influences the entire AVF lifespan.

To address these gaps, this single-centre retrospective cohort study applied Fine-Gray subdistribution hazard models to examine the independent associations of CAC and metabolic markers with both AVF functionality (the primary outcome) and post-functionality AVF failure (the secondary outcome) within the same patient cohort. We further evaluated model discrimination, calibration, and clinical utility through optimism-corrected C-index, calibration curves, and decision curve analysis, thereby providing a comprehensive assessment of whether preoperative static markers can reliably stratify risk across the entire AVF lifespan.

## 2 Methods

### 2.1 Design and population

This single-centre retrospective cohort study consecutively included 645 patients with ESKD who met the inclusion criteria and underwent first-time radiocephalic AVF creation at our institution between January 2021 and January 2024. All operations were performed by a single surgical team using a standardised anastomotic technique (end-to-side or side-to-side, according to intraoperative vascular anatomy). Patients with prior ipsilateral vascular access, active systemic infection, or incomplete baseline CT imaging were excluded. The study was approved by the institutional ethics committee, and the requirement for individual informed consent was waived because of the retrospective design and anonymised data handling. CT imaging, echocardiography, upper extremity vascular ultrasound, and laboratory sampling are all routinely performed as part of the preoperative workup after admission for AVF creation. Coronary artery calcium (CAC) scoring was performed on non-contrast, non-electrocardiogram (ECG)-gated chest CT images using an artificial intelligence-based software (CoronaryDoc, ShuKun Technology, Beijing, China). The software automatically quantified the Agatston score with a threshold of 130 Hounsfield units. All results were generated by the AI system without additional manual re-measurement. All CT examinations were performed using a standard low-dose non-contrast protocol (120 kVp, automatic tube current modulation, 2.0 mm slice thickness with 2.0 mm reconstruction interval) on a GE Optima CT680 Expert scanner (GE Healthcare, Chicago, IL, USA). The median interval between CT acquisition and AVF surgery was 4 days (IQR 3–7 days, range 2–10 days; *n* = 619). All 619 patients with complete CT data were scanned within 10 days before surgery; CT scans performed at outside hospitals were accepted if DICOM data were available and slice thickness was ≤2.5 mm. Two outcomes were analysed in the same cohort. The primary outcome was failure of AVF functionality, assessed from AVF creation in all 645 patients, with unassisted functionality, death and transplantation before functionality as competing events; patients lost to follow-up were censored. A secondary outcome was post-functionality AVF failure, assessed from the date of the first successful two-needle cannulation for a complete haemodialysis session among the 568 patients whose fistulas achieved successful functionality. Death and kidney transplantation were treated as competing events.

### 2.2 Outcome definitions

Functionality was defined as the first successful cannulation with both arterial and venous needles for a complete haemodialysis session, without ultrasound confirmation, and no fixed time window was applied. Cases that required radiological or surgical intervention before successful cannulation were not counted as successful functionality; they were included in the functionality-failure endpoint along with fistulas abandoned before cannulation. Unassisted functionality, death before functionality, and kidney transplantation before functionality were treated as competing events; patients lost to follow-up before functionality were censored.

Post-functionality AVF failure was defined as a study-specific composite outcome after successful functionality. Time zero was the date of the first successful two-needle cannulation for a complete haemodialysis session. The endpoint was the first occurrence of duplex ultrasound-confirmed access flow insufficiency (<300 mL/min), AVF thrombosis, or permanent access abandonment because of irreversible dysfunction. Death and kidney transplantation were treated as competing events. Because this composite endpoint incorporates ultrasound-defined flow insufficiency and access abandonment, it should not be interpreted as equivalent to standardized primary or functional primary patency definitions in vascular-access guidelines (1). We therefore use the descriptive term “post-functionality AVF failure” throughout this study.

### 2.3 Statistical analysis

Covariates comprised sex, age, hypertension, diabetes, mean arterial pressure, body mass index, radial artery and cephalic vein diameters, left ventricular diastolic dysfunction, ejection fraction, CAC score, haemoglobin, calcium, phosphate, HDL cholesterol, LDL cholesterol, triglycerides, C-reactive protein, serum albumin, dialysis vintage, and history of dialysis before first AVF use; assisted functionality was additionally included for the secondary outcome.

Collinearity was assessed by Spearman correlations (with particular focus on the CAC score) (Supplementary Table S3), variance inflation factors (VIF; Supplementary Table S4), and by refitting the models without C-reactive protein and without ejection fraction (Supplementary Tables S3–S5 and Supplementary Figure S5). Radial artery diameter was modelled using a 3-knot restricted cubic spline (RCS). Missing covariate values (all <5%) were imputed with 20 chained-equation imputations that included the event indicator and the Nelson-Aalen cumulative hazard, and estimates were combined by Rubin's rules.

Fine-Gray subdistribution hazard models were fitted for both stages. Continuous variables were modelled as restricted cubic splines or linear terms; the functional form of each continuous covariate is reported in Supplementary Table S6. Missing data (<5%) were imputed using multiple imputation by chained equations. Model performance was evaluated using optimism-corrected C-index, calibration curves, and decision curve analysis. Detailed statistical methods are provided in the Supplementary Material. All analyses were performed using R version 4.1.2. A two-sided *P*-value < 0.05 was considered statistically significant. This study was reported in accordance with the STROBE guidelines for observational studies.

## 3 Results

### 3.1 The primary outcome: functionality

A total of 645 patients met the inclusion criteria for the primary outcome (Figure 1). Functionality failure occurred in 139 patients (21.6%), comprising 93 fistulas that achieved functionality only after intervention and 46 fistulas abandoned before cannulation, whereas 475 fistulas (73.6%) achieved functionality without assistance. Nineteen patients had a competing event (death or transplantation) and 12 were lost to follow-up before functionality (Supplementary Table S1). Baseline characteristics are shown in Table 1. Patients with functionality failure were older (67.38 (11.13) vs. 59.18 (12.88) years, *P* < 0.001) and had a markedly higher CAC score (median 154.65 (8.69–583.00) vs. 0.00 (0.00–19.32) Agatston, *P* < 0.001) (Supplementary Figure S8).

**Figure 1:**
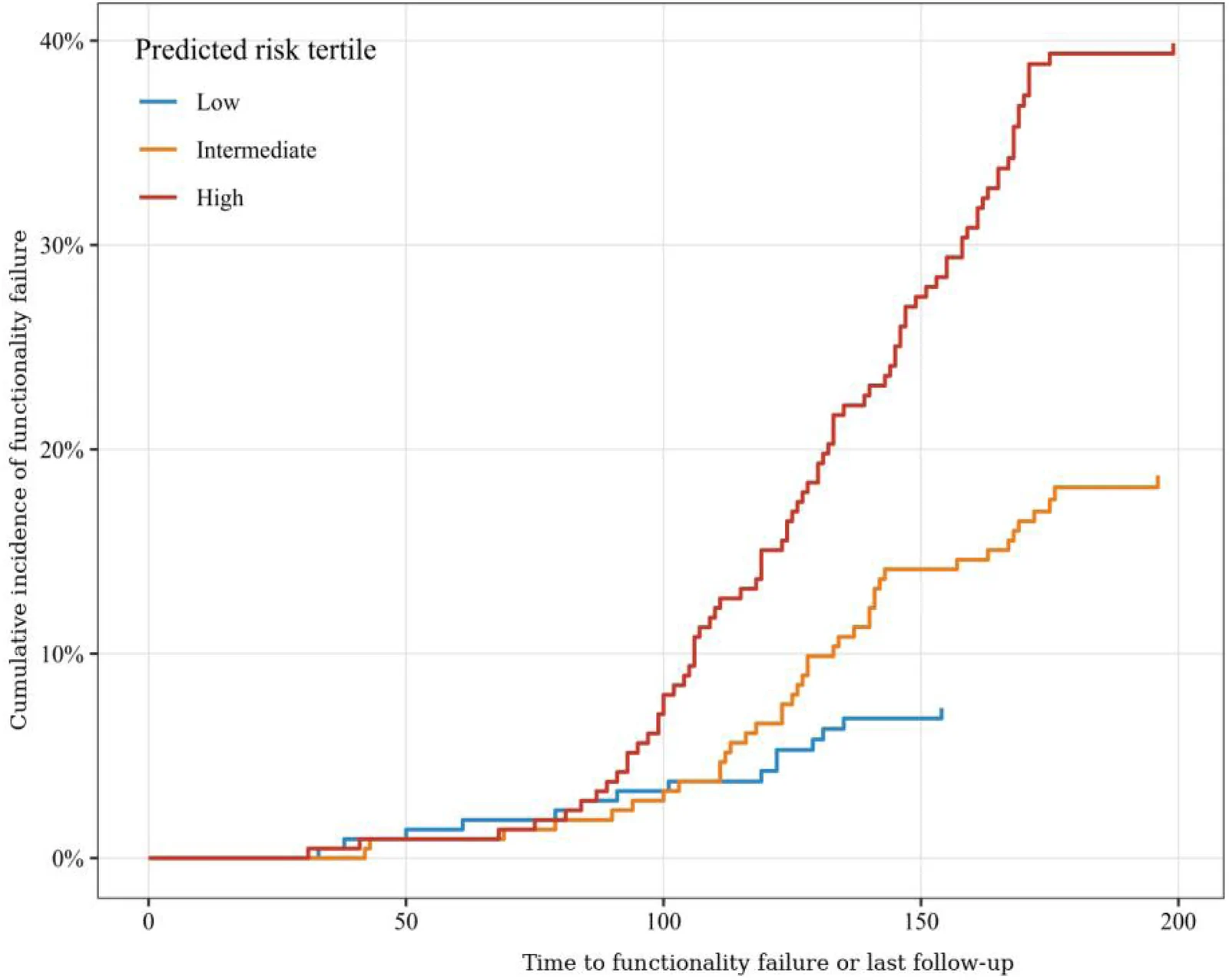
Study cohort flow diagram. Between January 2021 and January 2024, 645 patients with end-stage kidney disease who underwent first-time radiocephalic arteriovenous fistula (AVF) creation were included in the analysis of the primary outcome. Functionality failure occurred in 139 patients (21.6%; 93 after assisted functionality, 46 abandoned before cannulation), unassisted functionality in 475 (73.6%), competing events (death or transplantation) before functionality in 19, and 12 were lost to follow-up. Among 568 fistulas that achieved successful functionality, post-functionality AVF failure occurred in 277 (48.8%), competing events occurred in 75, and 216 were censored.

**Table 1:** Baseline characteristics of the study cohort according to the primary outcome (failure of arteriovenous fistula functionality).

Characteristic	Overall (*n* = 645)	functionality failure (*n* = 139)	Unassisted functionality (*n* = 475)	Missing, *n*	SMD	*P* value
Male sex, *n* (%)	427 (66.2)	90 (64.7)	314 (66.1)	0	0.03	0.767
Age, years	61.14 (12.89)	67.38 (11.13)	59.18 (12.88)	0	0.68	<0.001
Hypertension, *n* (%)	608 (94.3)	132 (95.0)	447 (94.1)	0	0.04	0.701
Diabetes mellitus, *n* (%)	272 (42.2)	74 (53.2)	185 (38.9)	0	0.29	0.003
Mean arterial pressure, mmHg	117.18 (18.03)	117.07 (17.71)	117.07 (18.32)	0	0.00	0.999
Body mass index, kg/m2	22.94 (3.69)	23.10 (3.61)	22.81 (3.69)	26	0.08	0.409
Radial artery diameter, mm	2.34 (0.41)	2.27 (0.38)	2.37 (0.41)	20	0.24	0.014
Cephalic vein diameter, mm	2.07 (0.55)	1.97 (0.57)	2.09 (0.54)	20	0.21	0.038
LV diastolic dysfunction, *n* (%)	285 (45.6)	78 (57.8)	198 (43.0)	20	0.30	0.002
LV ejection fraction, %	62.50 (7.96)	63.55 (8.30)	62.22 (7.78)	20	0.16	0.099
CAC score, Agatston	1.90 (0.00–106.23)	154.65 (8.69–583.00)	0.00 (0.00–19.32)	26	0.63	<0.001
Haemoglobin, g/L	85.58 (18.64)	85.97 (19.10)	85.36 (18.61)	1	0.03	0.739
Serum calcium, mmol/L	2.00 (0.24)	2.01 (0.21)	2.00 (0.25)	0	0.05	0.597
Serum phosphate, mmol/L	1.75 (0.50)	1.66 (0.45)	1.77 (0.51)	4	0.24	0.010
HDL cholesterol, mmol/L	1.06 (0.36)	1.07 (0.29)	1.06 (0.38)	8	0.04	0.695
LDL cholesterol, mmol/L	2.24 (0.93)	2.28 (1.01)	2.23 (0.91)	8	0.06	0.559
Triglycerides, mmol/L	1.26 (0.91–1.76)	1.23 (0.92–1.67)	1.27 (0.91–1.81)	8	0.14	0.525
C-reactive protein, mg/L	4.31 (0.64–36.72)	6.74 (1.29–39.41)	3.41 (0.54–31.26)	26	0.07	0.038
Serum albumin, g/L	34.90 (5.21)	34.67 (4.89)	35.06 (5.31)	26	0.08	0.433
Dialysis vintage at AVF creation, months	8.36 (1.05–19.39)	8.11 (1.20–16.49)	8.31 (1.02–19.92)	9	0.06	0.538
No dialysis before first AVF use, *n* (%)	19 (3.0)	1 (0.7)	18 (3.8)	9	0.21	0.091
Time from AVF creation to first use, days	77.00 (53.00–98.25)	-	-	77	-	-

Values are mean (SD), median (IQR) or *n* (%). The Overall column includes all 645 patients. The functionality failure and Unassisted functionality columns sum to 614; the remaining 31 patients comprise 19 competing events (death or transplantation before functionality) and 12 lost to follow-up before functionality, who are included in the Overall column but excluded from between-group comparisons.

In the multivariable Fine-Gray model (Table 2, Figure 2), each standard deviation increase in age (1.53 (1.22–1.93), *P* < 0.001) and in the CAC score (1.21 (1.06–1.38), *P* = 0.004), and the presence of left ventricular diastolic dysfunction (1.44 (1.02–2.04), *P* = 0.040), were independently associated with functionality failure. None of the newly available laboratory variables was associated with the outcome (haemoglobin 0.97 (0.80–1.18), *P* = 0.776; calcium 1.07 (0.88–1.29), *P* = 0.503; phosphate 0.93 (0.77–1.12), *P* = 0.441; HDL cholesterol 0.94 (0.79–1.13), *P* = 0.532; LDL cholesterol 1.12 (0.92–1.36), *P* = 0.263; triglycerides 0.87 (0.68–1.11), *P* = 0.271), nor were body mass index (1.02 (0.85–1.23), *P* = 0.799), diabetes (1.23 (0.81–1.88), *P* = 0.328) or C-reactive protein (0.99 (0.74–1.32), *P* = 0.946). Given the event-per-variable (EPV) ratio of approximately 6.3 in the fully adjusted model (139 events/22 covariates), we pre-specified a parsimonious model with 6 covariates (EPV ≈ 23). The parsimonious model confirmed the same independent predictors: age (SHR 1.56, 95% CI 1.25–1.94, *P* < 0.001), CAC score (SHR 1.21, 95% CI 1.07–1.36, *P* = 0.002), and left ventricular diastolic dysfunction (SHR 1.50, 95% CI 1.07–2.12, *P* = 0.020). The estimates for the two primary exposures were essentially identical across the fully adjusted, parsimonious, and minimally adjusted models (Table 2 and Supplementary Tables S18, S19), indicating that these associations are not an artefact of overfitting. Cumulative incidence curves stratified by tertile of predicted risk separated clearly (Gray test *P* < 0.001; Figure 3; Supplementary Table S12). Discrimination was 0.677 (95% CI 0.597–0.751) at 120 days and 0.746 (95% CI 0.7–0.79) at 180 days (Supplementary Table S10), and calibration was good across quintiles of predicted risk (maximum absolute difference 0.065; Supplementary Figure S1, Supplementary Table S9). After Benjamini–Hochberg false-discovery-rate (FDR) correction across all 22 covariates, age (P_BH = 0.004) and the CAC score (P_BH = 0.038) remained significant for functionality failure; left ventricular diastolic dysfunction was not retained as an independent predictor (P_BH = 0.24) and should be interpreted as hypothesis-generating. Notably, when applying the more conservative Holm correction for family-wise error rate (FWER) control, the CAC score yielded P_Holm = 0.073 (Supplementary Table S22), indicating that this association is significant under FDR control but does not reach the conventional threshold under FWER control. Readers should therefore interpret the CAC finding with appropriate caution.

**Table 2:** Multivariable fine-gray subdistribution hazard model for the primary outcome (failure of arteriovenous fistula functionality).

Variable	SHR (95% CI)	*P* value
Age, per 1 SD	1.53 (1.22–1.93)	<0.001
CAC score, per 1 SD	1.21 (1.06–1.38)	0.004
LV diastolic dysfunction	1.44 (1.02–2.04)	0.040
LV ejection fraction, per 1 SD	1.15 (0.94–1.41)	0.179
Cephalic vein diameter, per 1 SD	0.88 (0.72–1.07)	0.203
Mean arterial pressure, per 1 SD	1.13 (0.94–1.37)	0.203
No dialysis before first AVF use	0.27 (0.03–2.19)	0.222
LDL cholesterol, per 1 SD	1.12 (0.92–1.36)	0.263
Triglycerides, per 1 SD	0.87 (0.68–1.11)	0.271
Diabetes	1.23 (0.81–1.88)	0.328
Radial artery diameter, per 1 SD	0.83 (0.56–1.24)	0.366
Serum phosphate, per 1 SD	0.93 (0.77–1.12)	0.441
Serum calcium, per 1 SD	1.07 (0.88–1.29)	0.503
HDL cholesterol, per 1 SD	0.94 (0.79–1.13)	0.532
Male sex	1.07 (0.74–1.54)	0.723
Dialysis vintage, per 1 SD	0.97 (0.79–1.19)	0.768
Haemoglobin, per 1 SD	0.97 (0.80–1.18)	0.776
Body mass index, per 1 SD	1.02 (0.85–1.23)	0.799
Radial artery diameter (spline term)	0.97 (0.63–1.49)	0.879
Serum albumin, per 1 SD	1.02 (0.77–1.36)	0.882
C-reactive protein, per 1 SD	0.99 (0.74–1.32)	0.946
Hypertension	1.01 (0.46–2.21)	0.986

*n* = 645 with 139 events. Competing events were death or kidney transplantation before functionality (*n* = 19) and unassisted functionality (*n* = 475); patients lost to follow-up before functionality (*n* = 12) were censored. An SHR above 1 indicates a higher risk of functionality failure. Continuous variables are expressed per 1 SD increase. Radial artery diameter was modelled with a 3-knot restricted cubic spline; the spline term has no independent clinical interpretation. Missing covariate values (all <5%) were imputed with 20 chained-equation imputations and estimates were combined by Rubin's rules (Supplementary Table S2). AVF, arteriovenous fistula; CAC, coronary artery calcium; CI, confidence interval; HDL, high-density lipoprotein; IQR, interquartile range; LDL, low-density lipoprotein; LV, left ventricular; SD, standard deviation; SHR, subdistribution hazard ratio; SMD, standardised mean difference.

**Figure 2:**
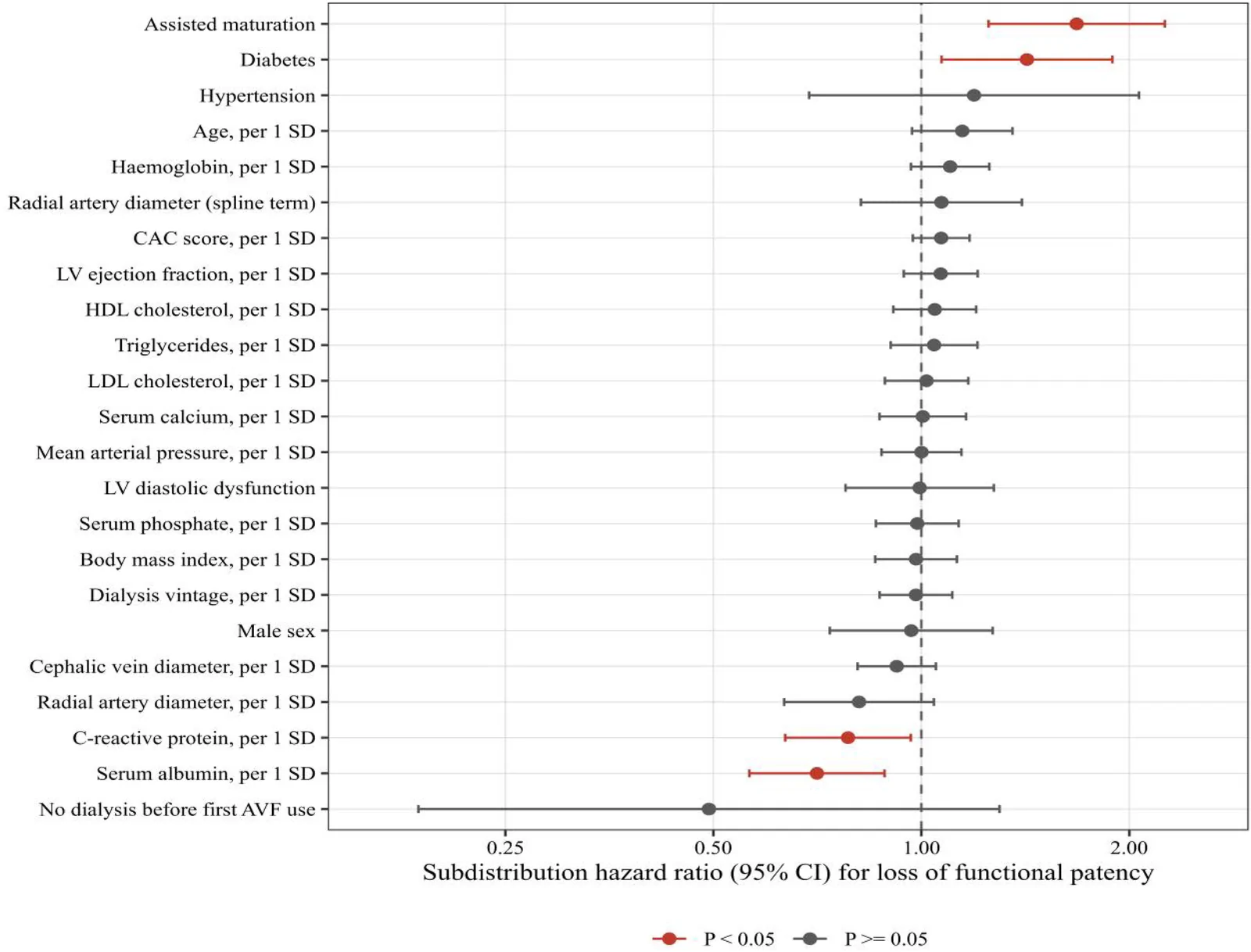
Forest plot of the multivariable fine-gray subdistribution hazard model for AVF functionality failure (the primary outcome) in 645 patients with 139 events. Competing events were unassisted functionality (*n* = 475), death or transplantation before functionality (*n* = 19); 12 patients were lost to follow-up. Forest plot of the multivariable Fine-Gray subdistribution hazard model for AVF functionality failure (the primary outcome) in 645 patients with 139 events. Squares represent subdistribution hazard ratios (SHR) and horizontal lines 95% confidence intervals (CI) for each covariate; the vertical dashed line indicates SHR = 1. Age (per 1 SD), coronary artery calcium (CAC) score (per 1 SD), and left ventricular diastolic dysfunction were independent predictors. Radial artery diameter was modelled with a 3-knot restricted cubic spline; the spline term has no independent clinical interpretation. Competing events were unassisted functionality (*n* = 475), death or transplantation before functionality (*n* = 19); 12 patients were lost to follow-up.

**Figure 3:**
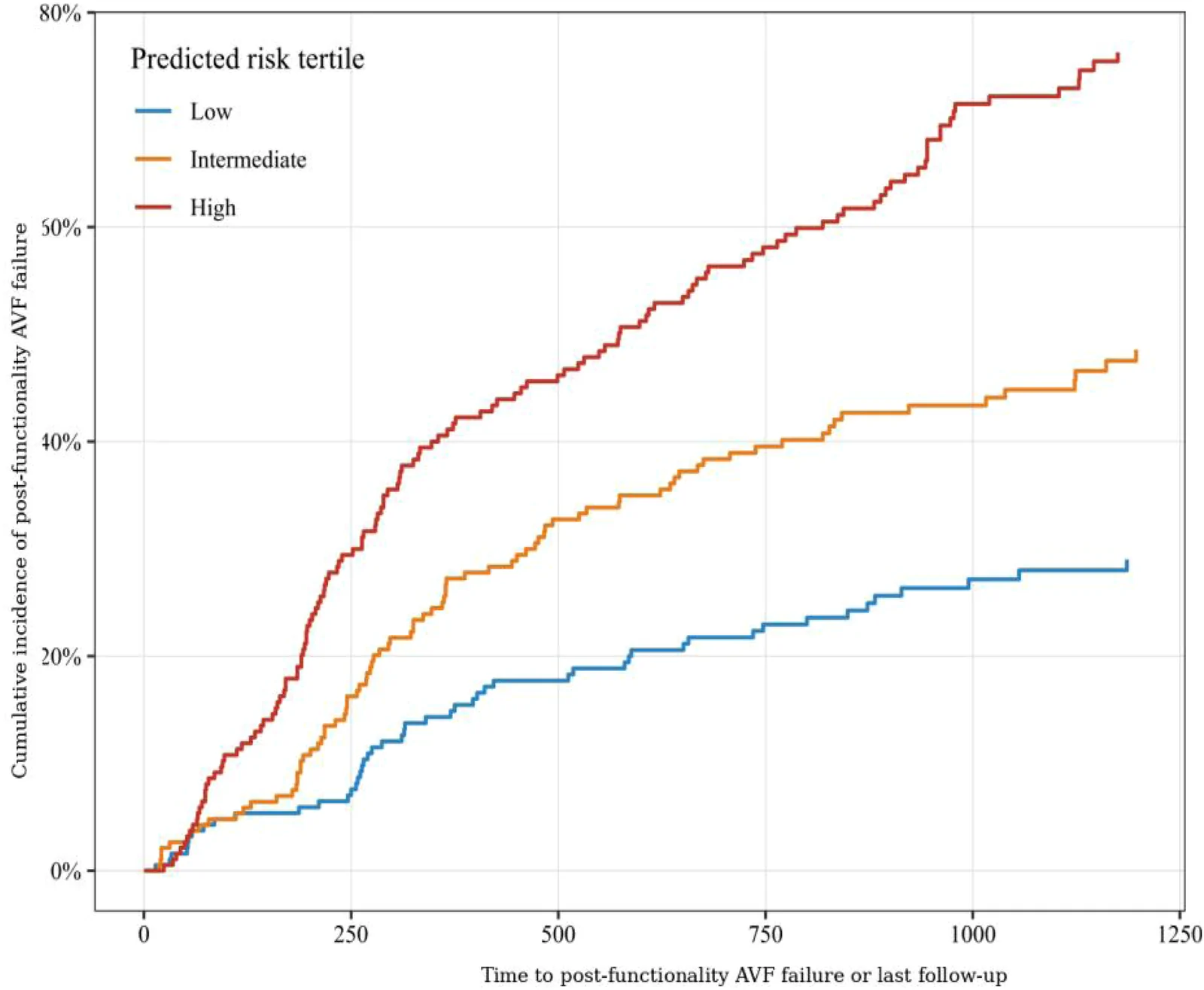
Cumulative incidence curves of AVF functionality failure stratified by tertiles of predicted risk. The 645 patients were divided into low-, intermediate-, and high-risk tertiles based on predicted risk of functionality failure from the multivariable Fine-Gray model. Cumulative incidence differed significantly across tertiles (Gray test *P* < 0.001). The low-risk group (*n* = 215) experienced 15 primary events, the intermediate-risk group (*n* = 215) 40 events, and the high-risk group (*n* = 215) 84 events. Competing events were death or kidney transplantation before functionality (*n* = 19) and unassisted functionality (*n* = 475); patients lost to follow-up (*n* = 12) were censored.

### 3.2 A secondary outcome: post-functionality AVF failure

Among the 568 patients whose fistulas achieved successful functionality, post-functionality AVF failure occurred in 277 patients (48.8%), with 75 competing events and 216 censored observations. The median time from successful functionality to post-functionality AVF failure was 328 days (IQR 194–670 days); median follow-up for the entire cohort was 1,055 days (IQR 465–1,327 days, approximately 2.9 years) (Supplementary Figure S12). Baseline characteristics are shown in Table 3. In the multivariable model (Table 4, Figure 4), assisted functionality was the strongest predictor (1.68 (1.25–2.25), *P* < 0.001), followed by diabetes (1.42 (1.07–1.89), *P* = 0.015); higher serum albumin (0.71 (0.56–0.88), *P* = 0.002) and, unexpectedly, higher C-reactive protein (0.78 (0.64–0.97), *P* = 0.022) were associated with a lower risk. Age (1.15 (0.97–1.35), *P* = 0.110) and the CAC score (1.07 (0.97–1.17), *P* = 0.171) were not significant when modelled per standard deviation; when the CAC score was modelled categorically, both strata carried a higher risk than a score of zero (1.58 (1.13–2.22), *P* = 0.008 for 1–100 and 1.55 (1.03–2.33), *P* = 0.038 for >100), consistent with the significant non-linearity of the CAC score in this outcome (*P* for non-linearity <0.001; Figure 5). Discrimination improved with follow-up (0.646 (95% CI 0.593–0.7) at 1 year, 0.677 (95% CI 0.632–0.725) at 2 years and 0.713 (95% CI 0.661–0.761) at 3 years; Supplementary Table S10), and calibration was acceptable (maximum absolute difference 0.059; Supplementary Figure S3; Supplementary Table S9). Cumulative incidence curves stratified by tertile of predicted risk separated clearly (Gray test *P* < 0.001; Figure 6). After Benjamini-Hochberg false-discovery-rate correction across all 22 covariates, assisted functionality (P_BH = 0.008) and serum albumin (P_BH = 0.022) remained significant for post-functionality AVF failure; diabetes and C-reactive protein were not retained as independent predictors (P_BH = 0.088) and should be interpreted with caution (Supplementary Table S23).

**Table 3:** Baseline characteristics of patients whose fistulas achieved successful functionality according to the secondary outcome (post-functionality AVF failure).

Characteristic	Overall (*n* = 568)	Post-functionality AVF failure (*n* = 277)	No post-functionality AVF failure (*n* = 291)	Missing, *n*	SMD	*P* value
Male sex, *n* (%)	373 (65.7)	179 (64.6)	194 (66.7)	0	0.04	0.608
Age, years	60.95 (12.93)	63.95 (12.29)	58.10 (12.89)	0	0.46	<0.001
Hypertension, *n* (%)	537 (94.5)	264 (95.3)	273 (93.8)	0	0.07	0.434
Diabetes mellitus, *n* (%)	236 (41.5)	138 (49.8)	98 (33.7)	0	0.33	<0.001
Mean arterial pressure, mmHg	117.15 (17.95)	117.27 (17.51)	117.04 (18.40)	0	0.01	0.879
Body mass index, kg/m2	22.81 (3.66)	22.92 (3.64)	22.71 (3.67)	23	0.06	0.490
Radial artery diameter, mm	2.34 (0.41)	2.28 (0.39)	2.40 (0.41)	18	0.31	<0.001
Cephalic vein diameter, mm	2.07 (0.54)	2.00 (0.52)	2.12 (0.57)	18	0.23	0.009
LV diastolic dysfunction, *n* (%)	255 (46.2)	136 (50.7)	119 (41.9)	16	0.18	0.037
LV ejection fraction, %	62.39 (7.91)	62.90 (8.17)	61.92 (7.64)	16	0.12	0.144
CAC score, Agatston	1.54 (0.00–97.09)	17.54 (0.00–391.04)	0.00 (0.00–9.12)	22	0.50	<0.001
Haemoglobin, g/L	85.77 (18.76)	86.65 (18.75)	84.94 (18.77)	0	0.09	0.278
Serum calcium, mmol/L	2.00 (0.25)	2.00 (0.23)	1.99 (0.26)	0	0.02	0.853
Serum phosphate, mmol/L	1.75 (0.50)	1.69 (0.49)	1.80 (0.51)	3	0.22	0.011
HDL cholesterol, mmol/L	1.06 (0.36)	1.07 (0.37)	1.05 (0.36)	8	0.05	0.580
LDL cholesterol, mmol/L	2.23 (0.93)	2.25 (0.99)	2.21 (0.86)	8	0.05	0.591
Triglycerides, mmol/L	1.26 (0.91–1.77)	1.27 (0.90–1.79)	1.25 (0.92–1.76)	8	0.00	0.852
C-reactive protein, mg/L	4.02 (0.61–32.36)	5.51 (1.15–38.75)	2.09 (0.42–23.65)	22	0.11	<0.001
Serum albumin, g/L	35.06 (5.21)	34.49 (4.97)	35.60 (5.38)	26	0.21	0.013
Dialysis vintage at AVF creation, months	7.95 (0.97–19.22)	7.46 (1.18–19.29)	8.31 (0.51–19.10)	1	0.02	0.960
No dialysis before first AVF use, *n* (%)	18 (3.2)	4 (1.4)	14 (4.8)	1	0.19	0.022
Time from AVF creation to first use, days	77.00 (53.00–98.25)	83.00 (55.00–105.00)	72.00 (53.00–92.00)	0	0.42	<0.001
Assisted functionality, *n* (%)	93 (16.4)	78 (28.2)	15 (5.2)	0	0.65	<0.001

*n* = 568 fistulas that achieved successful functionality. The Overall column includes all 568 patients. The post-functionality AVF failure column (*n* = 277) presents primary events. The No post-functionality AVF failure column (*n* = 291) is a composite of 216 patients alive with patent fistulas at last follow-up (censored) and 75 competing events (death or transplantation); these two subgroups are combined for baseline comparison but are analytically distinct in the survival analysis.

**Table 4:** Multivariable fine-gray subdistribution hazard model for the secondary outcome (post-functionality AVF failure).

Variable	SHR (95% CI)	*P* value
Assisted functionality	1.68 (1.25–2.25)	<0.001
Serum albumin, per 1 SD	0.71 (0.56–0.88)	0.002
Diabetes	1.42 (1.07–1.89)	0.015
C-reactive protein, per 1 SD	0.78 (0.64–0.97)	0.022
Radial artery diameter, per 1 SD	0.81 (0.63–1.04)	0.103
Age, per 1 SD	1.15 (0.97–1.35)	0.110
Haemoglobin, per 1 SD	1.10 (0.97–1.25)	0.149
No dialysis before first AVF use	0.49 (0.19–1.30)	0.152
CAC score, per 1 SD	1.07 (0.97–1.17)	0.171
Cephalic vein diameter, per 1 SD	0.92 (0.81–1.05)	0.218
LV ejection fraction, per 1 SD	1.07 (0.94–1.21)	0.304
HDL cholesterol, per 1 SD	1.05 (0.91–1.20)	0.526
Hypertension	1.19 (0.69–2.07)	0.531
Triglycerides, per 1 SD	1.04 (0.90–1.21)	0.565
Radial artery diameter (spline term)	1.07 (0.82–1.40)	0.625
Dialysis vintage, per 1 SD	0.98 (0.87–1.11)	0.771
Body mass index, per 1 SD	0.98 (0.86–1.13)	0.799
LDL cholesterol, per 1 SD	1.02 (0.89–1.17)	0.807
Male sex	0.97 (0.74–1.27)	0.807
Serum phosphate, per 1 SD	0.99 (0.86–1.13)	0.847
Serum calcium, per 1 SD	1.00 (0.87–1.16)	0.947
LV diastolic dysfunction	0.99 (0.78–1.27)	0.965
Mean arterial pressure, per 1 SD	1.00 (0.88–1.14)	0.994

*n* = 568 with 277 events and 75 competing events (death or kidney transplantation). Time zero was the date of the first successful two-needle cannulation for a complete haemodialysis session. An SHR above 1 indicates a higher risk of post-functionality AVF failure. Continuous variables are expressed per 1 SD increase. AVF, arteriovenous fistula; CAC, coronary artery calcium; CI, confidence interval; HDL, high-density lipoprotein; IQR, interquartile range; LDL, low-density lipoprotein; LV, left ventricular; SD, standard deviation; SHR, subdistribution hazard ratio; SMD, standardised mean difference.

**Figure 4:**
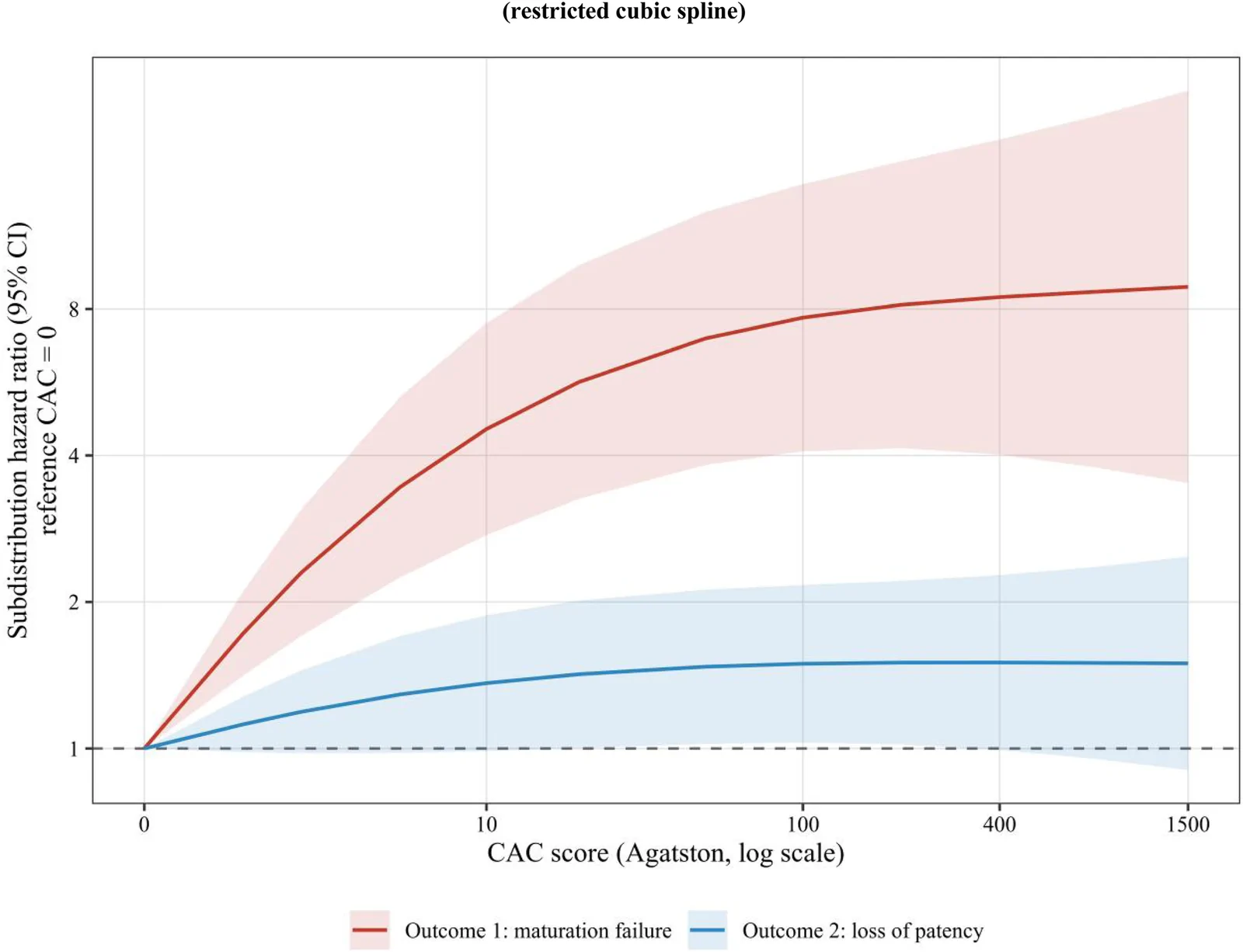
Forest plot of the multivariable fine-gray subdistribution hazard model for post-functionality AVF failure (the secondary outcome) in 568 patients with 277 events. Competing events were death or kidney transplantation (*n* = 75). Forest plot of the multivariable Fine-Gray subdistribution hazard model for post-functionality AVF failure (the secondary outcome) in 568 patients with 277 events. Squares represent subdistribution hazard ratios (SHR) and horizontal lines 95% confidence intervals (CI); the vertical dashed line indicates SHR = 1. Assisted functionality was the strongest predictor (SHR 1.68, 95% CI 1.25-2.25, *P* < 0.001), followed by diabetes, serum albumin, and C-reactive protein. Age and CAC score did not reach statistical significance when modelled per standard deviation. Competing events were death or kidney transplantation (*n* = 75).

**Figure 5:**
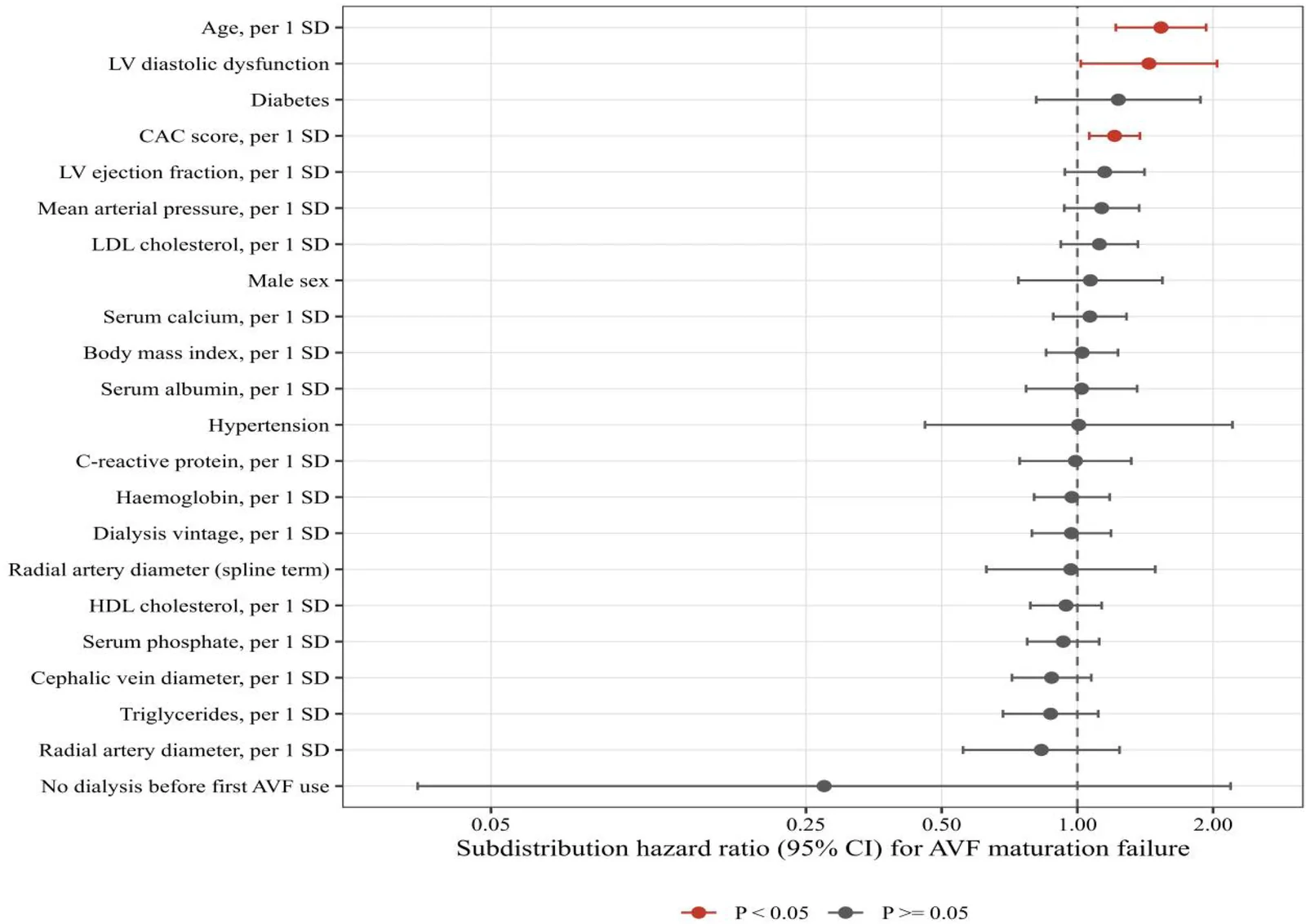
Dose-response relationship between coronary artery calcium score and post-functionality AVF failure (restricted cubic spline). In the multivariable Fine-Gray model for the secondary outcome, the CAC score showed a significant non-linear association with post-functionality AVF failure (*P* for non-linearity <0.001). The solid line represents the estimated subdistribution hazard ratio (SHR) and the shaded area the 95% confidence interval, with the reference set at CAC = 0. When modelled categorically, CAC scores of 1–100 and >100 vs. 0 yielded SHRs of 1.58 (1.13–2.22, *P* = 0.008) and 1.55 (1.03–2.33, *P* = 0.038), respectively.

**Figure 6:**
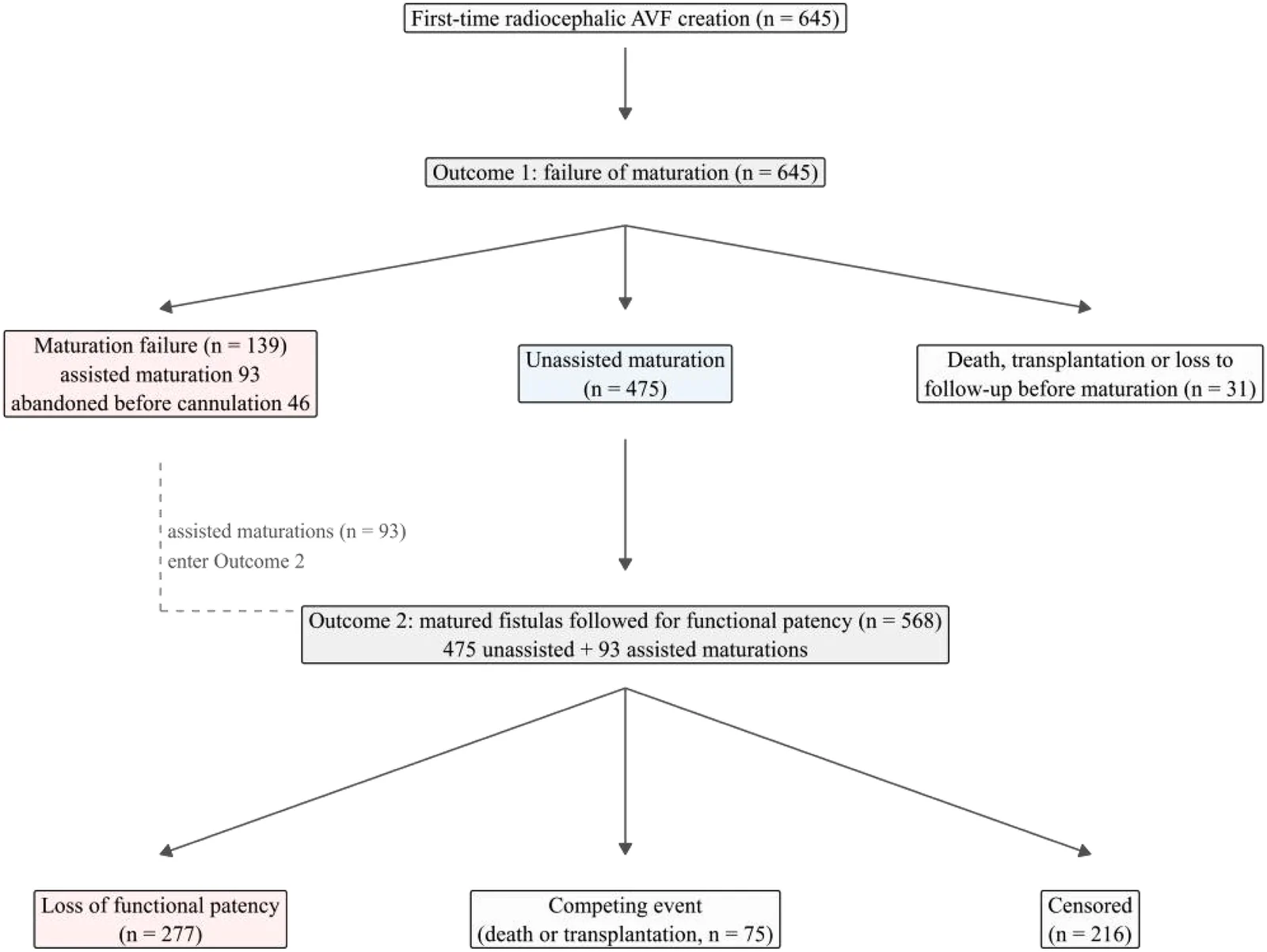
Cumulative incidence curves of post-functionality AVF failure stratified by tertiles of predicted risk. The 568 patients whose fistulas achieved successful functionality were divided into low-, intermediate-, and high-risk tertiles based on predicted risk of post-functionality AVF failure. Cumulative incidence differed significantly across tertiles (Gray test *P* < 0.001). The low-risk group (*n* = 190) experienced 52 primary events, the intermediate-risk group (*n* = 189) 88 events, and the high-risk group (*n* = 189) 137 events. Competing events were death (*n* = 70) or kidney transplantation (*n* = 5); censoring included loss to follow-up (*n* = 40) and patients alive with patent fistulas at last follow-up (*n* = 176).

## 4 Discussion

### 4.1 Significance of coronary artery calcium

A higher coronary artery calcium burden was independently associated with failure of AVF functionality (1.21 (1.06–1.38), *P* = 0.004 per standard deviation; 5.38 (3.02–9.58), *P* < 0.001 for a score of 1–100 and 6.61 (3.57–12.22), *P* < 0.001 for a score above 100, relative to a score of zero (Supplementary Figures S6, S9). These estimates were robust across model specifications (Supplementary Table S13 and Supplementary Figure S11). For post-functionality AVF failure the association was apparent only when the score was modelled categorically (1.58 (1.13–2.22), *P* = 0.008 and 1.55 (1.03–2.33), *P* = 0.038), consistent with the strongly skewed distribution of the score and with the formal evidence of non-linearity(Supplementary Figure S10). Two qualifications are important. First, the covariate most strongly correlated with the CAC score was age (Spearman rho = 0.522, *P* < 0.001), and residual confounding by age and by the overall burden of vascular disease cannot be excluded. Sensitivity analyses excluding C-reactive protein and ejection fraction yielded consistent estimates for the CAC score (Supplementary Table S13, Supplementary Figure S10). Second, the CAC score is an imaging surrogate of systemic calcification and not a measurement of the vessels used for the anastomosis (13). Histological studies of arterial specimens obtained at fistula creation have not demonstrated an association between medial microcalcification and functionality failure, stenosis or patency (14, 15). We therefore interpret the CAC score as a marker of the vascular substrate as a whole rather than as evidence that local calcification mechanically impairs remodelling. Any causal link between calcification and intimal hyperplasia in fistulas remains hypothetical, and fistula remodelling, which depends on shear-stress mediated outward vessel enlargement (16, 17), differs from atherosclerotic plaque progression.

A statistical caveat should be noted. The association between CAC score and functionality failure was significant after Benjamini–Hochberg FDR correction (P_BH = 0.038) but did not reach statistical significance after the more conservative Holm correction (P_Holm = 0.073, Supplementary Table S22). Given the exploratory nature of our multivariable models and the relatively modest event count, we considered FDR control appropriate for the primary analysis, but we acknowledge that this finding is not robust to the most stringent multiple-testing correction. Further studies with larger sample sizes are needed to confirm the independent prognostic value of CAC for AVF functionality.

### 4.2 Stage-specific effect of diabetes

Diabetes was associated with post-functionality AVF failure (1.42 (1.07–1.89), *P* = 0.015) but not with functionality failure (1.23 (0.81–1.88), *P* = 0.328), which is compatible with the view that hyperglycaemia-related neointimal hyperplasia requires prolonged exposure before it becomes flow-limiting (18, 19), whereas early remodelling is dominated by the mechanical and haemodynamic properties of the vessels themselves. Body mass index was not associated with either outcome (1.02 (0.85–1.23), *P* = 0.799 for functionality failure and 0.98 (0.86–1.13), *P* = 0.799 for loss of patency), and we no longer draw any inference about adiposity and fistula outcomes.

### 4.3 Prognostic significance of assisted functionality for post-functionality AVF failure

The strongest determinant of post-functionality AVF failure was the need for assistance to achieve functionality (1.68 (1.25–2.25), *P* < 0.001), and its effect increased with time since functionality (interaction with log-time 1.74 (1.17–2.58), *P* = 0.006; Supplementary Tables S7, S8). Fistulas that required endovascular or surgical intervention before they could be cannulated therefore behaved differently from those that achieved functionality without assistance, even after adjustment for age, diabetes, vessel diameters, calcification and nutritional and inflammatory markers. The need for early assistance appears to be a marker of limited vascular remodelling capacity that persists after the fistula has been made usable (20), and it identifies a group in whom intensified ultrasound surveillance after functionality is likely to be worthwhile. Sensitivity analysis adjusting for functionality window strengthened the association (SHR 2.08, 95% CI 1.34–3.25, Supplementary Table S15), suggesting that delayed functionality partially suppresses the true adverse effect of procedural intervention in the primary model; the estimates for diabetes, serum albumin, and C-reactive protein remained essentially unchanged. This variable also accounts for much of the improvement in discrimination relative to a model restricted to preoperative characteristics. The composite nature of the secondary outcome (flow insufficiency, thrombosis, or abandonment) merits consideration. Although our database did not permit mode-specific analyses, the predictors likely act through distinct mechanisms: diabetes promotes neointimal hyperplasia and progressive flow decline; assisted functionality signals a globally stenosis-prone vascular substrate; and hypoalbuminaemia impairs endothelial repair, predisposing to thrombosis and intervention intolerance. Coronary artery calcium, by contrast, appears to exert its primary effect during early remodelling (functionality) rather than during long-term maintenance, consistent with its role as a marker of arterial stiffness rather than of thrombotic risk.

### 4.4 Reverse association of C-reactive protein with post-functionality AVF failure

C-reactive protein was associated with a lower risk of post-functionality AVF failure (0.78 (0.64–0.97), *P* = 0.022), which is contrary to expectation (21, 22). The direction was the same in complete-case (0.80 (0.63–1.02), *P* = 0.066) and cause-specific (0.80 (0.65–0.99), *P* = 0.036) analyses (Supplementary Table S13), so it is not an artefact of imputation (22). A single preoperative measurement may reflect intercurrent illness rather than chronic inflammatory burden, and patients with a high C-reactive protein concentration were more likely to die, a competing event that lowers the subdistribution hazard of post-functionality AVF failure (21, 22). We report this association for completeness and draw no clinical inference from it (23).

### 4.5 Comparison with existing models

Previous AVF prediction models reported C-statistics of 0.72–0.79, around the commonly cited 0.75 threshold for clinical utility in individual risk stratification. Notably, these higher-performing models typically incorporated postoperative dynamic variables—such as early ultrasound flow volume, resistance index, or anastomotic angle—rather than relying exclusively on preoperative static markers (24). This study is the first to incorporate CAC into AVF outcome prediction; however, even after broadening the predictor set to include systemic calcification, preoperative static markers demonstrated limited predictive utility. The improvement in C-index over time in the secondary outcome (from 0.65 at 1 year to 0.71 at 3 years) suggests that longer follow-up captures more failure events but does not fundamentally overcome the limitations of baseline-only prediction (25). Future model improvement will require the integration of postoperative dynamic haemodynamic parameters, such as serial ultrasound flow measurements, dynamic blood pressure trajectories, and biomarker changes during the first 3 months after surgery (26).

### 4.6 Serum albumin and radial artery diameter

Higher serum albumin was independently associated with a lower risk of post-functionality AVF failure (SHR 0.71, 95% CI 0.56–0.88, *P* = 0.002), and the association was consistent in the parsimonious (0.72, 95% CI 0.58–0.89, *P* = 0.003), complete-case (0.71, 95% CI 0.55–0.91, *P* = 0.007) and cause-specific (0.75, 95% CI 0.61–0.93, *P* = 0.009) analyses (Supplementary Table S13, S20), indicating that it is not an artefact of imputation or of the modelling approach. This finding is consistent with prior literature (27). In ESKD patients, hypoalbuminaemia is a core manifestation of protein-energy wasting and can impair vascular repair through multiple mechanisms, including reduced nitric oxide bioavailability, exacerbated inflammation, and impaired endothelial progenitor cell function.

Radial artery diameter showed a protective trend in the main model (per 1 SD SHR 0.81, 95% CI 0.63–1.04, *P* = 0.103) that reached statistical significance in the parsimonious model (SHR 0.87, 95% CI 0.76–0.99, *P* = 0.039) (Supplementary Table S20). This discrepancy suggests that the association between radial artery diameter and post-functionality AVF failure may follow an approximately linear dose–response relationship, and that RCS decomposition in a modest sample size may have overfitted nonlinearity, thereby diluting the linear signal. Clinically, a larger radial artery diameter implies greater inflow volume and higher shear stress, both of which favour long-term fistula patency. These findings reinforce the importance of preoperative vascular mapping, particularly in patients with borderline vessel size.

### 4.7 Implications of low discrimination

A C-index in the range of 0.65–0.75 does not reflect model failure; rather, it reflects the inherent dynamic nature of AVF outcomes. Once created, AVF patency is dominated by postoperative haemodynamic feedback loops—shear stress, pulsatile stretch, repeated cannulation injury, and local inflammation—that cannot be anticipated from preoperative cross-sectional snapshots. Preoperative CAC, BMI, and diabetes capture only the baseline vascular substrate; they cannot predict the magnitude or direction of postoperative remodelling, which is shaped by patient-specific haemodynamic milieu, surgical technique, and cannulation practices. Decision curve analysis confirmed that, despite modest discrimination, the models conferred modest net benefit at intermediate threshold probabilities (15%–35%) (Supplementary Table S11; Supplementary Figures S2, S4), suggesting potential utility in population-level screening rather than individual prognostication.

From a pragmatic standpoint, the high-risk triad of advanced age, CAC >0, and diabetes identifies a subgroup of patients who may benefit from intensified postoperative ultrasound surveillance and early referral for vascular intervention. Conversely, a CAC score of zero in younger non-diabetic patients may possess negative predictive value, supporting a less intensive monitoring strategy and potentially reducing unnecessary healthcare utilisation. Future predictive breakthroughs should integrate four complementary directions: (1) intraoperative parameters such as immediate post-anastomosis flow and venous distension rate; (2) postoperative dynamic parameters such as serial ultrasound flow measurements and resistance index trajectories; (3) novel biomarkers reflecting endothelial function and inflammatory-fibrotic pathways; and (4) machine-learning algorithms capable of capturing high-order nonlinear interactions in multimodal data.

### 4.8 Limitations

This study has several limitations. First, it was conducted at a single centre and requires external validation. The correlation structure among all covariates is illustrated in Supplementary Figure S5. Second, intact parathyroid hormone and albumin-corrected calcium were not recorded, so the characterisation of mineral and bone disorder is incomplete. Third, the CAC score is a surrogate for systemic calcification rather than a measurement of the anastomosed vessels. Fourth, 26 patients (4%) had incomplete CT imaging and were excluded. If these patients represented a higher-risk subgroup with greater CAC burden, their exclusion would have biased the CAC-functionality association toward the null, making our estimates conservative. Fifth, functionality was defined by the first successful two-needle cannulation for a complete haemodialysis session, which depends in part on clinical scheduling; 245 of 568 fistulas (43.1%) were first used more than 12 weeks after creation (Supplementary Figure S7), and fistulas that were anatomically suitable for cannulation but had not yet been required for dialysis could not be identified separately. However, sensitivity analyses adjusting for functionality window (Supplementary Table S15) and restricting to fistulas first used within 12 weeks (Supplementary Table S16) or to fistulas with unassisted functionality (Supplementary Table S17) yielded materially unchanged estimates. The secondary outcome was a study-specific composite incorporating ultrasound-defined flow insufficiency, thrombosis, and access abandonment and should not be interpreted as equivalent to standardized patency endpoints. In addition, detailed information on the use of non-tunnelled or tunnelled central venous catheters before AVF creation, including the timing, sequence, type and duration of catheter use, could not be reliably retrieved from the retrospective database, precluding adjustment for this variable. Sixth, the analysis of functionality failure was based on 139 events and 22 covariates (approximately 6 events per variable (EPV)), so the fully adjusted model may be over-fitted; however, a pre-specified parsimonious model and minimally adjusted models are reported alongside it and gave consistent estimates for age and for the CAC score. Seventh, unassisted functionality was treated as a competing event because it precludes subsequent functionality failure; an analysis in which it is treated as censoring is provided in Supplementary Table S14. Eighth, the Agatston method was originally validated on 3.0 mm ECG-gated CT; our 2.0 mm non-gated protocol may yield modestly different absolute scores, though the rank-order correlation with cardiovascular risk is preserved. Because scoring was fully automated without routine human oversight, we cannot exclude rare misclassification of aortic valve or pericardial calcification as coronary calcium. Prior radiation therapy to the upper extremity or chest was not systematically recorded in the retrospective database and could not be reliably excluded; however, this is rare in the ESKD population undergoing first-time AVF creation at our institution.

## 5 Conclusion

Advanced age and a higher coronary artery calcium burden are independent risk factors for AVF functionality failure; for post-functionality AVF failure neither was independently significant per standard deviation (age *P* = 0.110; CAC score *P* = 0.171), the CAC score being associated with the outcome only when modelled categorically. Diabetes shows a stage-specific effect, promoting post-functionality AVF failure but not affecting functionality, whereas body mass index was not associated with either stage. Modest-to-moderate discrimination (C-index ≈0.65–0.75) suggests that preoperative static markers have limited standalone predictive utility. The integration of postoperative dynamic haemodynamic parameters is essential for future model improvement and precision surveillance in vascular access care.

## Data Availability

The raw data supporting the conclusions of this article will be made available by the authors, without undue reservation.
