# Supplementary material for: Coronary artery calcium burden and metabolic indices in relation to arteriovenous fistula functionality and post-functionality failure
Source: Front Surg. 2026 Sep 28;13:1916736. doi: 10.3389/fsurg.2026.1916736 (PMC13656915; doi:10.3389/fsurg.2026.1916736)
Supplement: Supplementary file 1 [file Supplementaryfile1.docx]

**Supplementary Tables**

**Supplementary** Table S1. Classification of outcomes and follow-up status.

| **Category** | **n** | **%** |
| --- | --- | --- |
| **Primary outcome (n = 645)** |  |  |
| Functionality failure total | 139 | 21.6 |
| assisted functionality | 93 | 14.4 |
| abandonment before cannulation | 46 | 7.1 |
| Unassisted functionality (success) | 475 | 73.6 |
| Competing events before functionality (death) | 16 | 2.5 |
| Competing events before functionality (transplantation) | 3 | 0.5 |
| Lost to follow-up before functionality | 12 | 1.9 |
| **Secondary outcome (n = 568)** |  |  |
| Post-functionality AVF failure | 277 | 48.8 |
| Competing event: death | 70 | 12.3 |
| Competing event: transplantation | 5 | 0.9 |
| Censored: lost to follow-up | 40 | 7.0 |
| Censored: alive and patent at last follow-up | 176 | 31.0 |
| Unassisted functionality | 475 | 83.6 |
| Assisted functionality | 93 | 16.4 |

Percentages are of the corresponding cohort.

**Supplementary** Table S2. Missing covariate data before multiple imputation.

| **Variable** | **Primary outcome, n** | **Primary outcome, %** | **Secondary outcome, n** | **Secondary outcome, %** |
| --- | --- | --- | --- | --- |
| Male sex | 0 | 0 | 0 | 0 |
| Hypertension | 0 | 0 | 0 | 0 |
| Diabetes | 0 | 0 | 0 | 0 |
| LV diastolic dysfunction | 20 | 3.1 | 16 | 2.8 |
| No dialysis before first AVF use | 9 | 1.4 | 1 | 0.2 |
| Assisted functionality | 77 | 11.9 | 0 | 0 |
| Age | 0 | 0 | 0 | 0 |
| Mean arterial pressure | 0 | 0 | 0 | 0 |
| Body mass index | 26 | 4 | 23 | 4 |
| Radial artery diameter | 20 | 3.1 | 18 | 3.2 |
| Cephalic vein diameter | 20 | 3.1 | 18 | 3.2 |
| LV ejection fraction | 20 | 3.1 | 16 | 2.8 |
| CAC score | 26 | 4 | 22 | 3.9 |
| Haemoglobin | 1 | 0.2 | 0 | 0 |
| Serum calcium | 0 | 0 | 0 | 0 |
| Serum phosphate | 4 | 0.6 | 3 | 0.5 |
| HDL cholesterol | 8 | 1.2 | 8 | 1.4 |
| LDL cholesterol | 8 | 1.2 | 8 | 1.4 |
| Triglycerides | 8 | 1.2 | 8 | 1.4 |
| C-reactive protein | 26 | 4 | 22 | 3.9 |
| Serum albumin | 26 | 4 | 26 | 4.6 |
| Dialysis vintage | 9 | 1.4 | 1 | 0.2 |

All covariates had less than 5% missing values. Twenty imputed data sets were generated by chained equations (predictive mean matching for continuous variables, logistic regression for binary variables), with the event indicator and the Nelson–Aalen cumulative hazard included as predictors. History of dialysis before first AVF use was imputed by predictive mean matching because it is almost perfectly collinear with a dialysis vintage of zero.

**Supplementary** Table S3. Correlation of candidate covariates with the coronary artery calcium score.

| **Cohort** | **Variable** | **n** | **Spearman rho** | **P value** | **Pearson r, log(1+CAC)** | **P value** |
| --- | --- | --- | --- | --- | --- | --- |
| Primary outcome cohort (n = 645) | Haemoglobin | 618 | 0.071 | 0.077 | 0.059 | 0.145 |
| Primary outcome cohort (n = 645) | Serum calcium | 619 | 0.016 | 0.695 | 0.028 | 0.489 |
| Primary outcome cohort (n = 645) | Serum phosphate | 615 | -0.152 | <0.001 | -0.162 | <0.001 |
| Primary outcome cohort (n = 645) | HDL cholesterol | 611 | 0.076 | 0.060 | 0.057 | 0.158 |
| Primary outcome cohort (n = 645) | LDL cholesterol | 611 | 0.014 | 0.731 | -0.001 | 0.979 |
| Primary outcome cohort (n = 645) | Triglycerides | 611 | -0.046 | 0.254 | -0.069 | 0.089 |
| Primary outcome cohort (n = 645) | C-reactive protein | 593 | 0.19 | <0.001 | 0.099 | 0.016 |
| Primary outcome cohort (n = 645) | Serum albumin | 594 | -0.142 | <0.001 | -0.132 | 0.001 |
| Primary outcome cohort (n = 645) | LV ejection fraction | 603 | 0.139 | <0.001 | 0.131 | 0.001 |
| Primary outcome cohort (n = 645) | Age | 619 | 0.522 | <0.001 | 0.581 | <0.001 |
| Primary outcome cohort (n = 645) | Body mass index | 595 | 0.039 | 0.342 | 0.003 | 0.937 |
| Primary outcome cohort (n = 645) | Mean arterial pressure | 619 | -0.043 | 0.283 | -0.063 | 0.118 |
| Primary outcome cohort (n = 645) | Radial artery diameter | 599 | 0.051 | 0.214 | 0.044 | 0.278 |
| Primary outcome cohort (n = 645) | Cephalic vein diameter | 599 | -0.057 | 0.165 | -0.049 | 0.229 |
| Primary outcome cohort (n = 645) | Dialysis vintage | 611 | -0.042 | 0.296 | -0.046 | 0.254 |
| Secondary outcome cohort (n = 568) | Haemoglobin | 546 | 0.088 | 0.040 | 0.077 | 0.074 |
| Secondary outcome cohort (n = 568) | Serum calcium | 546 | 0.035 | 0.410 | 0.04 | 0.351 |
| Secondary outcome cohort (n = 568) | Serum phosphate | 543 | -0.159 | <0.001 | -0.168 | <0.001 |
| Secondary outcome cohort (n = 568) | HDL cholesterol | 538 | 0.065 | 0.132 | 0.043 | 0.324 |
| Secondary outcome cohort (n = 568) | LDL cholesterol | 538 | 0.029 | 0.508 | 0.018 | 0.685 |
| Secondary outcome cohort (n = 568) | Triglycerides | 538 | -0.032 | 0.458 | -0.056 | 0.196 |
| Secondary outcome cohort (n = 568) | C-reactive protein | 524 | 0.192 | <0.001 | 0.106 | 0.015 |
| Secondary outcome cohort (n = 568) | Serum albumin | 521 | -0.135 | 0.002 | -0.128 | 0.003 |
| Secondary outcome cohort (n = 568) | LV ejection fraction | 532 | 0.151 | <0.001 | 0.136 | 0.002 |
| Secondary outcome cohort (n = 568) | Age | 546 | 0.517 | <0.001 | 0.581 | <0.001 |
| Secondary outcome cohort (n = 568) | Body mass index | 525 | 0.052 | 0.235 | 0.008 | 0.847 |
| Secondary outcome cohort (n = 568) | Mean arterial pressure | 546 | -0.025 | 0.559 | -0.044 | 0.306 |
| Secondary outcome cohort (n = 568) | Radial artery diameter | 528 | 0.052 | 0.229 | 0.042 | 0.332 |
| Secondary outcome cohort (n = 568) | Cephalic vein diameter | 528 | -0.046 | 0.295 | -0.039 | 0.375 |
| Secondary outcome cohort (n = 568) | Dialysis vintage | 545 | -0.054 | 0.205 | -0.062 | 0.147 |

Pearson correlations were computed on log(1 + CAC) because the CAC score is heavily right-skewed.

**Supplementary** Table S4. Variance inflation factors in the fully adjusted models.

| **Variable** | **Primary outcome** | **Secondary outcome** |
| --- | --- | --- |
| Radial artery diameter, per 1 SD | 6.01 | 5.49 |
| Radial artery diameter (spline term) | 5.98 | 5.36 |
| C-reactive protein, per 1 SD | 2.94 | 2.83 |
| Serum albumin, per 1 SD | 2.58 | 2.53 |
| Age, per 1 SD | 1.48 | 1.52 |
| Diabetes | 1.48 | 1.51 |
| Triglycerides, per 1 SD | 1.47 | 1.5 |
| CAC score, per 1 SD | 1.4 | 1.55 |
| HDL cholesterol, per 1 SD | 1.34 | 1.38 |
| LDL cholesterol, per 1 SD | 1.29 | 1.28 |
| Haemoglobin, per 1 SD | 1.28 | 1.29 |
| Mean arterial pressure, per 1 SD | 1.27 | 1.29 |
| Body mass index, per 1 SD | 1.2 | 1.21 |
| Serum phosphate, per 1 SD | 1.2 | 1.21 |
| Serum calcium, per 1 SD | 1.19 | 1.19 |
| Hypertension | 1.15 | 1.15 |
| Male sex | 1.15 | 1.15 |
| Cephalic vein diameter, per 1 SD | 1.15 | 1.18 |
| LV diastolic dysfunction | 1.1 | 1.12 |
| Dialysis vintage, per 1 SD | 1.07 | 1.07 |
| LV ejection fraction, per 1 SD | 1.07 | 1.07 |
| No dialysis before first AVF use | 1.07 | 1.08 |

Computed from the complete-case design matrix. The two radial artery diameter terms are the linear and spline components of the same variable and are collinear by construction; all other variance inflation factors were below 3.

**Supplementary** Table S5. Effect of removing C-reactive protein or left ventricular ejection fraction on the CAC estimate (complete-case models).

| **Outcome** | **Term removed** | **n** | **CAC in full model** | **CAC in reduced model** | **Change in SHR** |
| --- | --- | --- | --- | --- | --- |
| Primary outcome | C-reactive protein | 502 | 1.00 (0.85–1.18), P=0.963 | 1.03 (0.88–1.20), P=0.702 | +0.027 |
| Primary outcome | LV ejection fraction | 502 | 1.00 (0.85–1.18), P=0.963 | 1.00 (0.85–1.18), P=0.995 | -0.003 |
| Secondary outcome | C-reactive protein | 446 | 1.04 (0.93–1.17), P=0.501 | 1.04 (0.93–1.16), P=0.507 | -0.001 |
| Secondary outcome | LV ejection fraction | 446 | 1.04 (0.93–1.17), P=0.501 | 1.04 (0.93–1.17), P=0.493 | +0.001 |

**Supplementary** Table S6. Functional-form diagnostics for continuous covariates.

| **Outcome** | **Variable** | **n** | **Likelihood-ratio chi-square** | **P for non-linearity** | **Interpretation** |
| --- | --- | --- | --- | --- | --- |
| Primary outcome | Age | 645 | 0.01 | 0.914 | approximately linear |
| Primary outcome | Mean arterial pressure | 645 | 0.16 | 0.692 | approximately linear |
| Primary outcome | Body mass index | 619 | 0.03 | 0.867 | approximately linear |
| Primary outcome | Radial artery diameter | 625 | 0 | 0.980 | approximately linear |
| Primary outcome | Cephalic vein diameter | 625 | 0.32 | 0.571 | approximately linear |
| Primary outcome | LV ejection fraction | 625 | 0.02 | 0.879 | approximately linear |
| Primary outcome | CAC score | 619 | 1.28 | 0.258 | approximately linear |
| Primary outcome | Haemoglobin | 644 | 0.53 | 0.466 | approximately linear |
| Primary outcome | Serum calcium | 645 | 1.27 | 0.259 | approximately linear |
| Primary outcome | Serum phosphate | 641 | 0.5 | 0.479 | approximately linear |
| Primary outcome | HDL cholesterol | 637 | 5.93 | 0.015 | nonlinear |
| Primary outcome | LDL cholesterol | 637 | 0.54 | 0.464 | approximately linear |
| Primary outcome | Triglycerides | 637 | 0.51 | 0.477 | approximately linear |
| Primary outcome | C-reactive protein | 619 | 1.15 | 0.283 | approximately linear |
| Primary outcome | Serum albumin | 619 | 1.19 | 0.275 | approximately linear |
| Primary outcome | Dialysis vintage | 636 | 0.01 | 0.907 | approximately linear |
| Secondary outcome | Age | 568 | 0.37 | 0.546 | approximately linear |
| Secondary outcome | Mean arterial pressure | 568 | 0.39 | 0.535 | approximately linear |
| Secondary outcome | Body mass index | 545 | 0 | 0.976 | approximately linear |
| Secondary outcome | Radial artery diameter | 550 | 0 | 0.979 | approximately linear |
| Secondary outcome | Cephalic vein diameter | 550 | 0.68 | 0.410 | approximately linear |
| Secondary outcome | LV ejection fraction | 552 | 4.22 | 0.040 | nonlinear |
| Secondary outcome | CAC score | 546 | 11.94 | <0.001 | nonlinear |
| Secondary outcome | Haemoglobin | 568 | 0.01 | 0.924 | approximately linear |
| Secondary outcome | Serum calcium | 568 | 1.55 | 0.214 | approximately linear |
| Secondary outcome | Serum phosphate | 565 | 0.47 | 0.492 | approximately linear |
| Secondary outcome | HDL cholesterol | 560 | 0 | 0.981 | approximately linear |
| Secondary outcome | LDL cholesterol | 560 | 0.35 | 0.556 | approximately linear |
| Secondary outcome | Triglycerides | 560 | 0.54 | 0.460 | approximately linear |
| Secondary outcome | C-reactive protein | 546 | 3.36 | 0.067 | approximately linear |
| Secondary outcome | Serum albumin | 542 | 0.34 | 0.561 | approximately linear |
| Secondary outcome | Dialysis vintage | 567 | 1.49 | 0.222 | approximately linear |

Likelihood-ratio test comparing a linear term with a 3-knot restricted cubic spline (knots at the 10th, 50th and 90th percentiles) in cause-specific Cox models.

**Supplementary** Table S7. Proportional subdistribution hazards diagnostics.

| **Outcome** | **Variable** | **Residual slope versus log(time)** | **P value** | **Violation** |
| --- | --- | --- | --- | --- |
| Primary outcome | Age, per 1 SD | 0.4426 | 0.112 | no |
| Primary outcome | Serum albumin, per 1 SD | 0.4642 | 0.139 | no |
| Primary outcome | Radial artery diameter (spline term) | -0.3092 | 0.267 | no |
| Primary outcome | Radial artery diameter, per 1 SD | -0.4197 | 0.169 | no |
| Primary outcome | Body mass index, per 1 SD | -0.1177 | 0.712 | no |
| Primary outcome | Serum calcium, per 1 SD | -0.084 | 0.773 | no |
| Primary outcome | CAC score, per 1 SD | 0.6664 | 0.170 | no |
| Primary outcome | C-reactive protein, per 1 SD | -0.4541 | 0.159 | no |
| Primary outcome | Diabetes | 0.2898 | 0.061 | no |
| Primary outcome | Dialysis vintage, per 1 SD | -0.5322 | 0.103 | no |
| Primary outcome | LV ejection fraction, per 1 SD | -0.3813 | 0.283 | no |
| Primary outcome | Haemoglobin, per 1 SD | 0.0681 | 0.839 | no |
| Primary outcome | HDL cholesterol, per 1 SD | 0.036 | 0.897 | no |
| Primary outcome | Hypertension | 0.0121 | 0.860 | no |
| Primary outcome | LDL cholesterol, per 1 SD | -0.2226 | 0.542 | no |
| Primary outcome | LV diastolic dysfunction | 0.0748 | 0.661 | no |
| Primary outcome | Male sex | 0.0749 | 0.654 | no |
| Primary outcome | Mean arterial pressure, per 1 SD | 0.1582 | 0.630 | no |
| Primary outcome | No dialysis before first AVF use | -0.0905 | <0.001 | yes |
| Primary outcome | Serum phosphate, per 1 SD | -0.3491 | 0.281 | no |
| Primary outcome | Triglycerides, per 1 SD | -0.0194 | 0.940 | no |
| Primary outcome | Cephalic vein diameter, per 1 SD | -0.535 | 0.097 | no |
| Secondary outcome | Age, per 1 SD | 0.1625 | 0.017 | yes |
| Secondary outcome | Serum albumin, per 1 SD | -0.0211 | 0.759 | no |
| Secondary outcome | Radial artery diameter (spline term) | -0.1399 | 0.037 | yes |
| Secondary outcome | Radial artery diameter, per 1 SD | -0.1255 | 0.082 | no |
| Secondary outcome | Assisted functionality | 0.1277 | <0.001 | yes |
| Secondary outcome | Body mass index, per 1 SD | 0.0555 | 0.427 | no |
| Secondary outcome | Serum calcium, per 1 SD | 0.0061 | 0.928 | no |
| Secondary outcome | CAC score, per 1 SD | 0.2255 | 0.014 | yes |
| Secondary outcome | C-reactive protein, per 1 SD | 1e-04 | 0.999 | no |
| Secondary outcome | Diabetes | 0.0463 | 0.193 | no |
| Secondary outcome | Dialysis vintage, per 1 SD | 0.1285 | 0.086 | no |
| Secondary outcome | LV ejection fraction, per 1 SD | 0.0131 | 0.861 | no |
| Secondary outcome | Haemoglobin, per 1 SD | -0.1164 | 0.098 | no |
| Secondary outcome | HDL cholesterol, per 1 SD | 0.0243 | 0.734 | no |
| Secondary outcome | Hypertension | -0.0061 | 0.678 | no |
| Secondary outcome | LDL cholesterol, per 1 SD | -0.1528 | 0.043 | yes |
| Secondary outcome | LV diastolic dysfunction | 0.0261 | 0.459 | no |
| Secondary outcome | Male sex | 0.0198 | 0.565 | no |
| Secondary outcome | Mean arterial pressure, per 1 SD | -0.0332 | 0.647 | no |
| Secondary outcome | No dialysis before first AVF use | 0.0046 | 0.588 | no |
| Secondary outcome | Serum phosphate, per 1 SD | -0.1395 | 0.043 | yes |
| Secondary outcome | Triglycerides, per 1 SD | -0.0852 | 0.205 | no |
| Secondary outcome | Cephalic vein diameter, per 1 SD | -0.1017 | 0.117 | no |

**Supplementary** Table S8. Time-varying coefficients for covariates that violated the proportional subdistribution hazards assumption.

| **Outcome** | **Interaction term** | **SHR (95% CI)** | **P value** |
| --- | --- | --- | --- |
| Primary outcome | No dialysis before first AVF use × log(time) | 0.00 (0.00–2.55) | 0.070 |
| Secondary outcome | Age × log(time) | 1.02 (0.88–1.18) | 0.842 |
| Secondary outcome | Radial artery diameter (spline term) × log(time) | 0.95 (0.84–1.08) | 0.410 |
| Secondary outcome | Assisted functionality × log(time) | 1.74 (1.17–2.58) | 0.006 |
| Secondary outcome | CAC score × log(time) | 1.05 (0.93–1.19) | 0.430 |
| Secondary outcome | LDL cholesterol × log(time) | 0.89 (0.80–0.99) | 0.033 |
| Secondary outcome | Serum phosphate × log(time) | 0.91 (0.79–1.06) | 0.226 |

**Supplementary** Table S9. Calibration by quintile of predicted risk.

| **Outcome** | **Horizon, days** | **Quintile** | **n** | **Predicted** | **Observed** | **Absolute difference** |
| --- | --- | --- | --- | --- | --- | --- |
| Primary outcome | 90 | Q1 | 129 | 0.0081 | 0.027 | 0.0189 |
| Primary outcome | 90 | Q2 | 129 | 0.0149 | 0.0242 | 0.0093 |
| Primary outcome | 90 | Q3 | 129 | 0.0223 | 0.0221 | 2e-04 |
| Primary outcome | 90 | Q4 | 129 | 0.0339 | 0.0275 | 0.0064 |
| Primary outcome | 90 | Q5 | 129 | 0.0674 | 0.0465 | 0.0208 |
| Primary outcome | 120 | Q1 | 129 | 0.0244 | 0.0377 | 0.0133 |
| Primary outcome | 120 | Q2 | 129 | 0.0446 | 0.0723 | 0.0276 |
| Primary outcome | 120 | Q3 | 129 | 0.0666 | 0.032 | 0.0346 |
| Primary outcome | 120 | Q4 | 129 | 0.0999 | 0.0986 | 0.0013 |
| Primary outcome | 120 | Q5 | 129 | 0.1885 | 0.1854 | 0.0031 |
| Primary outcome | 180 | Q1 | 129 | 0.0676 | 0.0678 | 3e-04 |
| Primary outcome | 180 | Q2 | 129 | 0.1215 | 0.0987 | 0.0228 |
| Primary outcome | 180 | Q3 | 129 | 0.1775 | 0.131 | 0.0465 |
| Primary outcome | 180 | Q4 | 129 | 0.2579 | 0.3228 | 0.065 |
| Primary outcome | 180 | Q5 | 129 | 0.434 | 0.4471 | 0.0131 |
| Secondary outcome | 365.25 | Q1 | 114 | 0.1301 | 0.1535 | 0.0234 |
| Secondary outcome | 365.25 | Q2 | 113 | 0.1911 | 0.1813 | 0.0098 |
| Secondary outcome | 365.25 | Q3 | 114 | 0.2452 | 0.2484 | 0.0032 |
| Secondary outcome | 365.25 | Q4 | 113 | 0.3126 | 0.359 | 0.0464 |
| Secondary outcome | 365.25 | Q5 | 114 | 0.461 | 0.424 | 0.037 |
| Secondary outcome | 730.5 | Q1 | 114 | 0.2 | 0.1922 | 0.0078 |
| Secondary outcome | 730.5 | Q2 | 113 | 0.2884 | 0.3095 | 0.0211 |
| Secondary outcome | 730.5 | Q3 | 114 | 0.3631 | 0.3625 | 6e-04 |
| Secondary outcome | 730.5 | Q4 | 113 | 0.4515 | 0.4826 | 0.0311 |
| Secondary outcome | 730.5 | Q5 | 114 | 0.6247 | 0.6124 | 0.0123 |
| Secondary outcome | 1095.75 | Q1 | 114 | 0.2632 | 0.2402 | 0.023 |
| Secondary outcome | 1095.75 | Q2 | 113 | 0.3728 | 0.3621 | 0.0107 |
| Secondary outcome | 1095.75 | Q3 | 114 | 0.4613 | 0.4321 | 0.0292 |
| Secondary outcome | 1095.75 | Q4 | 113 | 0.5609 | 0.586 | 0.0251 |
| Secondary outcome | 1095.75 | Q5 | 114 | 0.7355 | 0.7949 | 0.0594 |

**Supplementary** Table S10. Discrimination (time-truncated concordance).

| **Outcome** | **Horizon, days** | **C-index** | **95% CI** | **Bootstrap resamples** |
| --- | --- | --- | --- | --- |
| Primary outcome | 90 | 0.566 | 0.436–0.701 | 500 |
| Primary outcome | 120 | 0.677 | 0.597–0.751 | 500 |
| Primary outcome | 180 | 0.746 | 0.7–0.79 | 500 |
| Secondary outcome | 365.25 | 0.646 | 0.593–0.7 | 500 |
| Secondary outcome | 730.5 | 0.677 | 0.632–0.725 | 500 |
| Secondary outcome | 1095.75 | 0.713 | 0.661–0.761 | 500 |

Concordance between the predicted cumulative incidence and observed events occurring before the horizon, with competing events treated as non-events; confidence intervals from 500 bootstrap resamples.

**Supplementary** Table S11. Decision curve analysis.

| **Outcome** | **Horizon, days** | **Threshold probability** | **Net benefit, model** | **Net benefit, treat all** |
| --- | --- | --- | --- | --- |
| Primary outcome | 90 | 0.05 | -6e-04 | -0.0222 |
| Primary outcome | 90 | 0.1 | 0 | -0.079 |
| Primary outcome | 90 | 0.15 | -7e-04 | -0.1425 |
| Primary outcome | 90 | 0.2 | -1e-04 | -0.2139 |
| Primary outcome | 90 | 0.25 | 4e-04 | -0.2948 |
| Primary outcome | 90 | 0.3 | 0 | -0.3873 |
| Primary outcome | 90 | 0.35 | 0 | -0.494 |
| Primary outcome | 90 | 0.4 | 0 | -0.6185 |
| Primary outcome | 90 | 0.45 | 0 | -0.7657 |
| Primary outcome | 90 | 0.5 | 0 | -0.9423 |
| Primary outcome | 120 | 0.05 | 0.039 | 0.0366 |
| Primary outcome | 120 | 0.1 | 0.0116 | -0.0169 |
| Primary outcome | 120 | 0.15 | 0.0111 | -0.0767 |
| Primary outcome | 120 | 0.2 | 0.004 | -0.144 |
| Primary outcome | 120 | 0.25 | 8e-04 | -0.2203 |
| Primary outcome | 120 | 0.3 | -0.0016 | -0.3074 |
| Primary outcome | 120 | 0.35 | -0.0032 | -0.408 |
| Primary outcome | 120 | 0.4 | -0.0032 | -0.5254 |
| Primary outcome | 120 | 0.45 | -0.0025 | -0.664 |
| Primary outcome | 120 | 0.5 | -0.0017 | -0.8304 |
| Primary outcome | 180 | 0.05 | 0.1747 | 0.174 |
| Primary outcome | 180 | 0.1 | 0.1357 | 0.1281 |
| Primary outcome | 180 | 0.15 | 0.11 | 0.0768 |
| Primary outcome | 180 | 0.2 | 0.0936 | 0.0191 |
| Primary outcome | 180 | 0.25 | 0.0622 | -0.0462 |
| Primary outcome | 180 | 0.3 | 0.0399 | -0.121 |
| Primary outcome | 180 | 0.35 | 0.0358 | -0.2072 |
| Primary outcome | 180 | 0.4 | 0.022 | -0.3078 |
| Primary outcome | 180 | 0.45 | 0.0181 | -0.4267 |
| Primary outcome | 180 | 0.5 | 0.0185 | -0.5694 |
| Secondary outcome | 365.25 | 0.05 | 0.2335 | 0.2353 |
| Secondary outcome | 365.25 | 0.1 | 0.1902 | 0.1928 |
| Secondary outcome | 365.25 | 0.15 | 0.1481 | 0.1453 |
| Secondary outcome | 365.25 | 0.2 | 0.118 | 0.0919 |
| Secondary outcome | 365.25 | 0.25 | 0.078 | 0.0314 |
| Secondary outcome | 365.25 | 0.3 | 0.0503 | -0.0378 |
| Secondary outcome | 365.25 | 0.35 | 0.022 | -0.1177 |
| Secondary outcome | 365.25 | 0.4 | -0.0025 | -0.2108 |
| Secondary outcome | 365.25 | 0.45 | -0.0125 | -0.3209 |
| Secondary outcome | 365.25 | 0.5 | -0.0071 | -0.453 |
| Secondary outcome | 730.5 | 0.05 | 0.3611 | 0.3611 |
| Secondary outcome | 730.5 | 0.1 | 0.3233 | 0.3256 |
| Secondary outcome | 730.5 | 0.15 | 0.2839 | 0.286 |
| Secondary outcome | 730.5 | 0.2 | 0.2475 | 0.2413 |
| Secondary outcome | 730.5 | 0.25 | 0.2044 | 0.1908 |
| Secondary outcome | 730.5 | 0.3 | 0.1724 | 0.133 |
| Secondary outcome | 730.5 | 0.35 | 0.117 | 0.0663 |
| Secondary outcome | 730.5 | 0.4 | 0.099 | -0.0115 |
| Secondary outcome | 730.5 | 0.45 | 0.0792 | -0.1035 |
| Secondary outcome | 730.5 | 0.5 | 0.045 | -0.2138 |
| Secondary outcome | 1095.75 | 0.05 | 0.4608 | 0.4608 |
| Secondary outcome | 1095.75 | 0.1 | 0.4291 | 0.4309 |
| Secondary outcome | 1095.75 | 0.15 | 0.3949 | 0.3974 |
| Secondary outcome | 1095.75 | 0.2 | 0.3584 | 0.3597 |
| Secondary outcome | 1095.75 | 0.25 | 0.3232 | 0.3171 |
| Secondary outcome | 1095.75 | 0.3 | 0.2838 | 0.2683 |
| Secondary outcome | 1095.75 | 0.35 | 0.2413 | 0.212 |
| Secondary outcome | 1095.75 | 0.4 | 0.2081 | 0.1463 |
| Secondary outcome | 1095.75 | 0.45 | 0.1632 | 0.0687 |
| Secondary outcome | 1095.75 | 0.5 | 0.154 | -0.0244 |

**Supplementary** Table S12. Risk tertiles defined by the multivariable models.

| **Outcome** | **Risk tertile** | **n** | **Primary events** | **Competing events** |
| --- | --- | --- | --- | --- |
| Primary outcome | Low | 215 | 15 | 196 |
| Primary outcome | Intermediate | 215 | 40 | 172 |
| Primary outcome | High | 215 | 84 | 126 |
| Secondary outcome | Low | 190 | 52 | 42 |
| Secondary outcome | Intermediate | 189 | 88 | 22 |
| Secondary outcome | High | 189 | 137 | 11 |

Gray test across tertiles: Primary outcome, event type 1: chi-square = 35.412, df = 2, P <0.001; Primary outcome, event type 2: chi-square = 56.014, df = 2, P <0.001; Secondary outcome, event type 1: chi-square = 79.533, df = 2, P <0.001; Secondary outcome, event type 2: chi-square = 25.681, df = 2, P <0.001.

**Supplementary** Table S13. Sensitivity analyses.

| **Outcome** | **Model** | **Variable** | **Estimate (95% CI)** | **P value** |
| --- | --- | --- | --- | --- |
| Primary outcome | CAC unadjusted | CAC score, per 1 SD | 1.44 (1.31–1.58) | <0.001 |
| Primary outcome | CAC + age only | CAC score, per 1 SD | 1.25 (1.12–1.40) | <0.001 |
| Primary outcome | CAC + age only | Age, per 1 SD | 1.56 (1.26–1.93) | <0.001 |
| Primary outcome | Parsimonious model | CAC score, per 1 SD | 1.21 (1.07–1.36) | 0.002 |
| Primary outcome | Parsimonious model | Age, per 1 SD | 1.55 (1.23–1.94) | <0.001 |
| Primary outcome | Parsimonious model | Diabetes | 1.27 (0.85–1.91) | 0.248 |
| Primary outcome | Parsimonious model | Serum albumin, per 1 SD | 0.97 (0.74–1.28) | 0.822 |
| Primary outcome | Parsimonious model | C-reactive protein, per 1 SD | 0.94 (0.71–1.25) | 0.690 |
| Primary outcome | CAC as log(1+CAC) | log(1+CAC), per 1 SD | 2.40 (1.80–3.19) | <0.001 |
| Primary outcome | CAC as categories | CAC 1-100 (vs 0) | 5.38 (3.02–9.58) | <0.001 |
| Primary outcome | CAC as categories | CAC >100 (vs 0) | 6.61 (3.57–12.22) | <0.001 |
| Primary outcome | Complete-case (full model) | CAC score, per 1 SD | 1.25 (1.09–1.44) | 0.001 |
| Primary outcome | Complete-case (full model) | Age, per 1 SD | 1.62 (1.26–2.09) | <0.001 |
| Primary outcome | Complete-case (full model) | Diabetes | 1.07 (0.67–1.71) | 0.778 |
| Primary outcome | Complete-case (full model) | Serum albumin, per 1 SD | 1.04 (0.75–1.42) | 0.826 |
| Primary outcome | Complete-case (full model) | C-reactive protein, per 1 SD | 1.15 (0.85–1.55) | 0.370 |
| Primary outcome | Cause-specific Cox (HR) | CAC score, per 1 SD | 0.97 (0.83–1.13) | 0.701 |
| Primary outcome | Cause-specific Cox (HR) | Age, per 1 SD | 0.98 (0.74–1.31) | 0.904 |
| Primary outcome | Cause-specific Cox (HR) | Diabetes | 1.08 (0.71–1.65) | 0.707 |
| Primary outcome | Cause-specific Cox (HR) | Serum albumin, per 1 SD | 1.18 (0.87–1.58) | 0.283 |
| Primary outcome | Cause-specific Cox (HR) | C-reactive protein, per 1 SD | 1.42 (1.04–1.94) | 0.030 |
| Secondary outcome | CAC unadjusted | CAC score, per 1 SD | 1.25 (1.17–1.33) | <0.001 |
| Secondary outcome | CAC + age only | CAC score, per 1 SD | 1.15 (1.07–1.25) | <0.001 |
| Secondary outcome | CAC + age only | Age, per 1 SD | 1.25 (1.09–1.44) | 0.002 |
| Secondary outcome | Parsimonious model | CAC score, per 1 SD | 1.07 (0.98–1.17) | 0.137 |
| Secondary outcome | Parsimonious model | Age, per 1 SD | 1.15 (0.98–1.35) | 0.084 |
| Secondary outcome | Parsimonious model | Diabetes | 1.38 (1.05–1.82) | 0.020 |
| Secondary outcome | Parsimonious model | Serum albumin, per 1 SD | 0.72 (0.58–0.89) | 0.003 |
| Secondary outcome | Parsimonious model | C-reactive protein, per 1 SD | 0.81 (0.66–0.99) | 0.036 |
| Secondary outcome | CAC as log(1+CAC) | log(1+CAC), per 1 SD | 1.19 (0.99–1.43) | 0.061 |
| Secondary outcome | CAC as categories | CAC 1-100 (vs 0) | 1.58 (1.13–2.22) | 0.008 |
| Secondary outcome | CAC as categories | CAC >100 (vs 0) | 1.55 (1.03–2.33) | 0.038 |
| Secondary outcome | Complete-case (full model) | CAC score, per 1 SD | 1.04 (0.93–1.17) | 0.501 |
| Secondary outcome | Complete-case (full model) | Age, per 1 SD | 1.09 (0.91–1.31) | 0.347 |
| Secondary outcome | Complete-case (full model) | Diabetes | 1.47 (1.06–2.03) | 0.021 |
| Secondary outcome | Complete-case (full model) | Serum albumin, per 1 SD | 0.71 (0.55–0.91) | 0.007 |
| Secondary outcome | Complete-case (full model) | C-reactive protein, per 1 SD | 0.80 (0.63–1.02) | 0.066 |
| Secondary outcome | Cause-specific Cox (HR) | CAC score, per 1 SD | 1.05 (0.94–1.18) | 0.382 |
| Secondary outcome | Cause-specific Cox (HR) | Age, per 1 SD | 1.18 (1.00–1.38) | 0.049 |
| Secondary outcome | Cause-specific Cox (HR) | Diabetes | 1.40 (1.05–1.87) | 0.021 |
| Secondary outcome | Cause-specific Cox (HR) | Serum albumin, per 1 SD | 0.75 (0.61–0.93) | 0.009 |
| Secondary outcome | Cause-specific Cox (HR) | C-reactive protein, per 1 SD | 0.80 (0.65–0.99) | 0.036 |
| Primary outcome | Unassisted functionality censored (letter spec) | CAC score, per 1 SD | 1.00 (0.87–1.15) | 0.967 |
| Primary outcome | Unassisted functionality censored (letter spec) | Age, per 1 SD | 1.05 (0.78–1.40) | 0.757 |
| Primary outcome | Unassisted functionality censored (letter spec) | Diabetes | 1.03 (0.70–1.51) | 0.895 |
| Primary outcome | Unassisted functionality censored (letter spec) | Serum albumin, per 1 SD | 1.26 (0.95–1.67) | 0.105 |
| Primary outcome | Unassisted functionality censored (letter spec) | C-reactive protein, per 1 SD | 1.34 (1.00–1.78) | 0.051 |
| Primary outcome | Unassisted functionality censored (letter spec) | LV diastolic dysfunction | 1.52 (1.07–2.16) | 0.019 |
| Primary outcome | Unassisted functionality censored (letter spec) | HDL cholesterol, per 1 SD | 0.80 (0.63–1.00) | 0.048 |
| Primary outcome | CAC unadjusted, censoring spec | CAC score, per 1 SD | 1.06 (0.95–1.17) | 0.286 |
| Primary outcome | CAC unadjusted, censoring spec | log(1+CAC), per 1 SD | 1.26 (1.06–1.49) | 0.009 |
| Primary outcome | Logistic regression (OR) | CAC score, per 1 SD | 1.56 (1.22–1.99) | <0.001 |
| Primary outcome | Logistic regression (OR) | Age, per 1 SD | 1.60 (1.20–2.12) | 0.001 |
| Primary outcome | Logistic regression (OR) | Diabetes | 1.33 (0.81–2.18) | 0.258 |
| Primary outcome | Logistic regression (OR) | Serum albumin, per 1 SD | 1.02 (0.71–1.46) | 0.907 |
| Primary outcome | Logistic regression (OR) | C-reactive protein, per 1 SD | 0.92 (0.63–1.33) | 0.643 |
| Primary outcome | Logistic regression (OR) | LV diastolic dysfunction | 1.42 (0.92–2.19) | 0.109 |
| Primary outcome | Logistic regression (OR) | HDL cholesterol, per 1 SD | 0.96 (0.75–1.22) | 0.730 |

Estimates are subdistribution hazard ratios unless otherwise stated; the logistic model reports odds ratios and the cause-specific model reports hazard ratios. In the model labelled 'unassisted functionality censored' unassisted functionality is treated as censoring rather than as a competing event.

**Supplementary** Table S14. Primary outcome with unassisted functionality treated as censoring (alternative competing-risk specification).

| **Variable** | **SHR (95% CI)** | **P value** |
| --- | --- | --- |
| LV diastolic dysfunction | 1.52 (1.07–2.16) | 0.019 |
| HDL cholesterol, per 1 SD | 0.80 (0.63–1.00) | 0.048 |
| LV ejection fraction, per 1 SD | 1.17 (1.00–1.38) | 0.051 |
| C-reactive protein, per 1 SD | 1.34 (1.00–1.78) | 0.051 |
| Serum albumin, per 1 SD | 1.26 (0.95–1.67) | 0.105 |
| Serum phosphate, per 1 SD | 0.89 (0.75–1.05) | 0.170 |
| No dialysis before first AVF use | 3.74 (0.32–43.78) | 0.293 |
| Haemoglobin, per 1 SD | 0.91 (0.76–1.11) | 0.363 |
| Triglycerides, per 1 SD | 0.88 (0.66–1.18) | 0.406 |
| Hypertension | 1.44 (0.59–3.51) | 0.425 |
| Cephalic vein diameter, per 1 SD | 0.92 (0.75–1.13) | 0.427 |
| Serum calcium, per 1 SD | 1.08 (0.89–1.32) | 0.431 |
| Dialysis vintage, per 1 SD | 1.09 (0.88–1.34) | 0.447 |
| Body mass index, per 1 SD | 0.96 (0.79–1.17) | 0.658 |
| Radial artery diameter, per 1 SD | 0.91 (0.59–1.41) | 0.681 |
| Radial artery diameter (spline term) | 1.08 (0.67–1.74) | 0.752 |
| Age, per 1 SD | 1.05 (0.78–1.40) | 0.757 |
| Mean arterial pressure, per 1 SD | 0.98 (0.80–1.20) | 0.871 |
| Diabetes | 1.03 (0.70–1.51) | 0.895 |
| CAC score, per 1 SD | 1.00 (0.87–1.15) | 0.967 |
| Male sex | 0.99 (0.67–1.46) | 0.974 |
| LDL cholesterol, per 1 SD | 1.00 (0.83–1.20) | 0.999 |

In this specification all associations are attenuated towards the null, including the unadjusted CAC estimate, although the median CAC score differed markedly between patients with functionality failure and patients with unassisted functionality (Table 1). Censoring patients at unassisted functionality implies that they remain at risk of functionality failure, which is not possible; the analysis is therefore presented as a sensitivity analysis only.

**Supplementary** Table S15. Fine–Gray model for post-functionality AVF failure (secondary outcome), additionally adjusted for the functionality window (n = 568, 277 events).

| **Covariate** | **SHR (95% CI)** | **P value** |
| --- | --- | --- |
| Assisted functionality | 2.08 (1.34–3.25) | 0.001 |
| Age (per 1 SD) | 1.15 (0.97–1.36) | 0.105 |
| Male sex | 0.95 (0.73–1.25) | 0.729 |
| Hypertension | 1.17 (0.67–2.04) | 0.576 |
| Diabetes | 1.44 (1.08–1.91) | 0.012 |
| Mean arterial pressure (per 1 SD) | 0.99 (0.86–1.13) | 0.867 |
| Body mass index (per 1 SD) | 0.99 (0.87–1.14) | 0.921 |
| Radial artery diameter (per 1 SD) | 0.81 (0.62–1.06) | 0.119 |
| Cephalic vein diameter (per 1 SD) | 0.93 (0.81–1.06) | 0.255 |
| LV diastolic dysfunction | 0.99 (0.77–1.27) | 0.948 |
| Ejection fraction (per 1 SD) | 1.06 (0.94–1.20) | 0.356 |
| CAC score (per 1 SD) | 1.07 (0.97–1.19) | 0.158 |
| Haemoglobin (per 1 SD) | 1.10 (0.96–1.25) | 0.171 |
| Calcium (per 1 SD) | 1.00 (0.87–1.16) | 0.963 |
| Phosphate (per 1 SD) | 0.99 (0.86–1.14) | 0.905 |
| HDL cholesterol (per 1 SD) | 1.05 (0.91–1.20) | 0.507 |
| LDL cholesterol (per 1 SD) | 1.02 (0.89–1.18) | 0.746 |
| Triglycerides (per 1 SD) | 1.04 (0.90–1.20) | 0.616 |
| C-reactive protein (per 1 SD) | 0.77 (0.62–0.95) | 0.013 |
| Serum albumin (per 1 SD) | 0.70 (0.56–0.88) | 0.002 |
| Dialysis vintage (per 1 SD) | 0.99 (0.87–1.14) | 0.933 |
| Dialysis before first AVF use | 0.98 (0.71–1.35) | 0.884 |
| Functionality window (per 1 SD) | 0.90 (0.76–1.08) | 0.275 |

*SHR, subdistribution hazard ratio; CI, confidence interval; LV, left ventricular; CAC, coronary artery calcium. Continuous covariates are expressed per 1 SD. Radial artery diameter was modelled with a 3-knot restricted cubic spline; the spline term (no independent interpretation) is omitted. Estimates pooled across 20 multiple imputations by Rubin’s rules.*

The functionality window itself was not independently associated with post-functionality AVF failure (SHR 0.90, 95% CI 0.76–1.08, P = 0.275), and the estimates for assisted functionality, diabetes, serum albumin and C-reactive protein were essentially unchanged.

**Supplementary** Table S16. Fine–Gray model for post-functionality AVF failure restricted to fistulas first used within 12 weeks (n = 323, 140 events).

| **Covariate** | **SHR (95% CI)** | **P value** |
| --- | --- | --- |
| Age (per 1 SD) | 1.19 (0.97–1.48) | 0.100 |
| Male sex | 0.83 (0.57–1.21) | 0.337 |
| Hypertension | 1.21 (0.49–3.00) | 0.676 |
| Diabetes | 1.45 (0.96–2.20) | 0.079 |
| Mean arterial pressure (per 1 SD) | 0.96 (0.80–1.16) | 0.680 |
| Body mass index (per 1 SD) | 1.07 (0.88–1.29) | 0.491 |
| Radial artery diameter (per 1 SD) | 0.75 (0.52–1.08) | 0.118 |
| Cephalic vein diameter (per 1 SD) | 0.96 (0.79–1.17) | 0.687 |
| LV diastolic dysfunction | 0.93 (0.65–1.33) | 0.687 |
| Ejection fraction (per 1 SD) | 1.10 (0.88–1.37) | 0.396 |
| CAC score (per 1 SD) | 1.23 (0.96–1.57) | 0.106 |
| Haemoglobin (per 1 SD) | 1.12 (0.92–1.37) | 0.260 |
| Calcium (per 1 SD) | 1.07 (0.87–1.32) | 0.523 |
| Phosphate (per 1 SD) | 1.11 (0.88–1.39) | 0.386 |
| HDL cholesterol (per 1 SD) | 1.01 (0.82–1.23) | 0.945 |
| LDL cholesterol (per 1 SD) | 0.99 (0.79–1.23) | 0.897 |
| Triglycerides (per 1 SD) | 1.01 (0.85–1.21) | 0.883 |
| C-reactive protein (per 1 SD) | 0.90 (0.62–1.29) | 0.552 |
| Serum albumin (per 1 SD) | 0.78 (0.52–1.17) | 0.227 |
| Dialysis vintage (per 1 SD) | 1.03 (0.87–1.22) | 0.715 |
| Dialysis before first AVF use | 1.07 (0.66–1.73) | 0.795 |

*SHR, subdistribution hazard ratio; CI, confidence interval; LV, left ventricular; CAC, coronary artery calcium. Continuous covariates are expressed per 1 SD. Radial artery diameter was modelled with a 3-knot restricted cubic spline; the spline term (no independent interpretation) is omitted. Estimates pooled across 20 multiple imputations by Rubin’s rules.*

**Supplementary** Table S17.Fine–Gray model for post-functionality AVF failure restricted to fistulas with unassisted functionality (n = 475, 199 events).

| **Covariate** | **SHR (95% CI)** | **P value** |
| --- | --- | --- |
| Age (per 1 SD) | 1.17 (0.98–1.39) | 0.090 |
| Male sex | 0.93 (0.68–1.28) | 0.654 |
| Hypertension | 1.16 (0.59–2.28) | 0.660 |
| Diabetes | 1.45 (1.04–2.03) | 0.030 |
| Mean arterial pressure (per 1 SD) | 1.04 (0.89–1.22) | 0.632 |
| Body mass index (per 1 SD) | 1.01 (0.85–1.19) | 0.924 |
| Radial artery diameter (per 1 SD) | 0.76 (0.55–1.05) | 0.095 |
| Cephalic vein diameter (per 1 SD) | 0.89 (0.76–1.04) | 0.135 |
| LV diastolic dysfunction | 0.99 (0.73–1.35) | 0.968 |
| Ejection fraction (per 1 SD) | 1.09 (0.93–1.27) | 0.275 |
| CAC score (per 1 SD) | 1.18 (1.00–1.39) | 0.055 |
| Haemoglobin (per 1 SD) | 1.06 (0.90–1.25) | 0.487 |
| Calcium (per 1 SD) | 1.05 (0.89–1.25) | 0.546 |
| Phosphate (per 1 SD) | 0.97 (0.81–1.16) | 0.768 |
| HDL cholesterol (per 1 SD) | 1.07 (0.91–1.25) | 0.414 |
| LDL cholesterol (per 1 SD) | 1.04 (0.87–1.25) | 0.678 |
| Triglycerides (per 1 SD) | 1.04 (0.89–1.21) | 0.643 |
| C-reactive protein (per 1 SD) | 0.79 (0.59–1.05) | 0.100 |
| Serum albumin (per 1 SD) | 0.72 (0.54–0.96) | 0.023 |
| Dialysis vintage (per 1 SD) | 1.05 (0.90–1.22) | 0.558 |
| Dialysis before first AVF use | 0.89 (0.60–1.33) | 0.571 |

*SHR, subdistribution hazard ratio; CI, confidence interval; LV, left ventricular; CAC, coronary artery calcium. Continuous covariates are expressed per 1 SD. Radial artery diameter was modelled with a 3-knot restricted cubic spline; the spline term (no independent interpretation) is omitted. Estimates pooled across 20 multiple imputations by Rubin’s rules.*

Directions were preserved across both restrictions; diabetes and serum albumin remained significant among fistulas with unassisted functionality, and the per-SD CAC association, null in the full cohort, was borderline in this subgroup (SHR 1.18, 95% CI 1.00–1.39, P = 0.055). Overall, differences in first-use timing did not materially confound the reported associations.

**Supplementary** Table S18. Parsimonious Fine–Gray model for AVF functionality failure (Primary outcome; 6 covariates, EPV ≈ 23).

| **Covariate** | **SHR (95% CI)** | **P value** |
| --- | --- | --- |
| Age (per 1 SD) | 1.56 (1.25–1.94) | <0.001 |
| Male sex | 1.03 (0.73–1.45) | 0.884 |
| Diabetes | 1.25 (0.87–1.78) | 0.228 |
| Radial artery diameter (per 1 SD) | 0.80 (0.67–0.96) | 0.014 |
| LV diastolic dysfunction | 1.50 (1.07–2.12) | 0.020 |
| CAC score (per 1 SD) | 1.21 (1.07–1.36) | 0.002 |

*SHR, subdistribution hazard ratio; CI, confidence interval; CAC, coronary artery calcium. Continuous covariates are expressed per 1 SD; radial artery diameter modelled linearly. Estimates pooled across 20 multiple imputations by Rubin’s rules.*

**Supplementary** Table S19. Minimally adjusted association of the CAC score with functionality failure (primary outcome).

| **Covariate** | **SHR (95% CI)** | **P value** |
| --- | --- | --- |
| Age (per 1 SD) | 1.56 (1.26–1.93) | <0.001 |
| Male sex | 0.99 (0.70–1.39) | 0.952 |
| CAC score (per 1 SD) | 1.25 (1.12–1.40) | <0.001 |

*SHR, subdistribution hazard ratio; CI, confidence interval; CAC, coronary artery calcium. Continuous covariates are expressed per 1 SD; radial artery diameter modelled linearly. Estimates pooled across 20 multiple imputations by Rubin’s rules.*

Age (SHR 1.56, 95% CI 1.25–1.94) and the CAC score (SHR 1.21, 95% CI 1.07–1.36) in the parsimonious model, and the CAC score in the minimally adjusted model (SHR 1.25, 95% CI 1.12–1.40), were essentially identical to the fully adjusted estimates, indicating that these associations are not an artefact of over-fitting the 22-covariate model.

**Supplementary** Table S20.Parsimonious Fine–Gray model for post-functionality AVF failure (Secondary outcome; 8 covariates, EPV ≈ 35).

| **Covariate** | **SHR (95% CI)** | **P value** |
| --- | --- | --- |
| Assisted functionality | 1.77 (1.34–2.35) | <0.001 |
| Age (per 1 SD) | 1.18 (1.01–1.37) | 0.038 |
| Male sex | 0.92 (0.72–1.18) | 0.515 |
| Diabetes | 1.46 (1.11–1.91) | 0.006 |
| Radial artery diameter (per 1 SD) | 0.86 (0.76–0.98) | 0.024 |
| CAC score (per 1 SD) | 1.06 (0.96–1.16) | 0.261 |
| C-reactive protein (per 1 SD) | 0.81 (0.66–0.98) | 0.029 |
| Serum albumin (per 1 SD) | 0.74 (0.61–0.91) | 0.004 |

*SHR, subdistribution hazard ratio; CI, confidence interval; CAC, coronary artery calcium. Continuous covariates are expressed per 1 SD; radial artery diameter modelled linearly. Estimates pooled across 20 multiple imputations by Rubin’s rules.*

**Supplementary** Table S21.Minimally adjusted association of the CAC score with post-functionality AVF failure (secondary outcome).

| **Covariate** | **SHR (95% CI)** | **P value** |
| --- | --- | --- |
| Assisted functionality | 1.92 (1.47–2.51) | <0.001 |
| Age (per 1 SD) | 1.18 (1.02–1.36) | 0.029 |
| Male sex | 0.95 (0.75–1.21) | 0.682 |
| CAC score (per 1 SD) | 1.09 (1.00–1.18) | 0.043 |

*SHR, subdistribution hazard ratio; CI, confidence interval; CAC, coronary artery calcium. Continuous covariates are expressed per 1 SD; radial artery diameter modelled linearly. Estimates pooled across 20 multiple imputations by Rubin’s rules.*

Assisted functionality, diabetes and serum albumin remained stable; radial artery diameter reached significance when modelled linearly (SHR 0.86, 95% CI 0.76–0.98), consistent with the pre-specified parsimonious result reported in the paper (0.87, 0.76–0.99). We recommend reporting the parsimonious coefficients in the main text as requested.

**Supplementary** Table S22.Multivariable Fine–Gray model for AVF functionality failure (primary outcome) with multiplicity-adjusted P values.

| **Covariate** | **SHR (95% CI)** | **P value** | **P (BH-FDR)** | **P (Holm)** |
| --- | --- | --- | --- | --- |
| Age (per 1 SD) | 1.55 (1.23–1.95) | <0.001 | 0.004 | 0.004 |
| Male sex | 1.05 (0.73–1.51) | 0.794 | 0.905 | 1.000 |
| Hypertension | 1.02 (0.47–2.23) | 0.959 | 0.959 | 1.000 |
| Diabetes | 1.25 (0.82–1.90) | 0.305 | 0.640 | 1.000 |
| Mean arterial pressure (per 1 SD) | 1.12 (0.93–1.36) | 0.240 | 0.640 | 1.000 |
| Body mass index (per 1 SD) | 1.04 (0.87–1.25) | 0.642 | 0.899 | 1.000 |
| Radial artery diameter (per 1 SD) | 0.80 (0.66–0.98) | 0.028 | 0.197 | 0.533 |
| Cephalic vein diameter (per 1 SD) | 0.90 (0.73–1.10) | 0.287 | 0.640 | 1.000 |
| LV diastolic dysfunction | 1.43 (1.01–2.03) | 0.045 | 0.239 | 0.820 |
| Ejection fraction (per 1 SD) | 1.14 (0.93–1.40) | 0.193 | 0.640 | 1.000 |
| CAC score (per 1 SD) | 1.21 (1.07–1.38) | 0.004 | 0.038 | 0.073 |
| Haemoglobin (per 1 SD) | 0.98 (0.81–1.19) | 0.817 | 0.905 | 1.000 |
| Calcium (per 1 SD) | 1.07 (0.88–1.28) | 0.509 | 0.809 | 1.000 |
| Phosphate (per 1 SD) | 0.92 (0.76–1.11) | 0.383 | 0.670 | 1.000 |
| HDL cholesterol (per 1 SD) | 0.94 (0.79–1.13) | 0.540 | 0.809 | 1.000 |
| LDL cholesterol (per 1 SD) | 1.12 (0.92–1.37) | 0.263 | 0.640 | 1.000 |
| Triglycerides (per 1 SD) | 0.87 (0.68–1.11) | 0.252 | 0.640 | 1.000 |
| C-reactive protein (per 1 SD) | 0.98 (0.74–1.30) | 0.873 | 0.917 | 1.000 |
| Serum albumin (per 1 SD) | 1.03 (0.78–1.37) | 0.818 | 0.905 | 1.000 |
| Dialysis vintage (per 1 SD) | 0.95 (0.76–1.20) | 0.691 | 0.905 | 1.000 |
| Dialysis before first AVF use | 1.25 (0.77–2.01) | 0.368 | 0.670 | 1.000 |

*SHR, subdistribution hazard ratio; CI, confidence interval. Continuous covariates per 1 SD; radial artery diameter modelled linearly. P (BH-FDR), Benjamini–Hochberg false-discovery-rate–adjusted P value; P (Holm), Holm-adjusted P value, across all covariates in the model.*

**Supplementary** Table S23. Multivariable Fine–Gray model for post-functionality AVF failure (secondary outcome) with multiplicity-adjusted P values.

| **Covariate** | **SHR (95% CI)** | **P value** | **P (BH-FDR)** | **P (Holm)** |
| --- | --- | --- | --- | --- |
| Assisted functionality | 1.71 (1.28–2.30) | <0.001 | 0.008 | 0.008 |
| Age (per 1 SD) | 1.15 (0.97–1.36) | 0.099 | 0.362 | 1.000 |
| Male sex | 0.95 (0.73–1.25) | 0.732 | 0.982 | 1.000 |
| Hypertension | 1.18 (0.68–2.04) | 0.564 | 0.982 | 1.000 |
| Diabetes | 1.43 (1.07–1.90) | 0.014 | 0.088 | 0.279 |
| Mean arterial pressure (per 1 SD) | 1.00 (0.87–1.14) | 0.959 | 0.982 | 1.000 |
| Body mass index (per 1 SD) | 0.99 (0.86–1.14) | 0.908 | 0.982 | 1.000 |
| Radial artery diameter (per 1 SD) | 0.86 (0.75–0.99) | 0.033 | 0.147 | 0.600 |
| Cephalic vein diameter (per 1 SD) | 0.92 (0.81–1.05) | 0.234 | 0.573 | 1.000 |
| LV diastolic dysfunction | 0.98 (0.77–1.26) | 0.891 | 0.982 | 1.000 |
| Ejection fraction (per 1 SD) | 1.07 (0.94–1.21) | 0.303 | 0.667 | 1.000 |
| CAC score (per 1 SD) | 1.07 (0.97–1.18) | 0.182 | 0.501 | 1.000 |
| Haemoglobin (per 1 SD) | 1.10 (0.97–1.25) | 0.149 | 0.468 | 1.000 |
| Calcium (per 1 SD) | 1.00 (0.87–1.16) | 0.968 | 0.982 | 1.000 |
| Phosphate (per 1 SD) | 0.99 (0.86–1.13) | 0.864 | 0.982 | 1.000 |
| HDL cholesterol (per 1 SD) | 1.05 (0.91–1.20) | 0.515 | 0.982 | 1.000 |
| LDL cholesterol (per 1 SD) | 1.02 (0.89–1.18) | 0.730 | 0.982 | 1.000 |
| Triglycerides (per 1 SD) | 1.04 (0.90–1.20) | 0.635 | 0.982 | 1.000 |
| C-reactive protein (per 1 SD) | 0.77 (0.63–0.95) | 0.016 | 0.088 | 0.304 |
| Serum albumin (per 1 SD) | 0.70 (0.56–0.88) | 0.002 | 0.022 | 0.042 |
| Dialysis vintage (per 1 SD) | 1.00 (0.87–1.15) | 0.982 | 0.982 | 1.000 |
| Dialysis before first AVF use | 0.97 (0.70–1.35) | 0.871 | 0.982 | 1.000 |

*SHR, subdistribution hazard ratio; CI, confidence interval. Continuous covariates per 1 SD; radial artery diameter modelled linearly. P (BH-FDR), Benjamini–Hochberg false-discovery-rate–adjusted P value; P (Holm), Holm-adjusted P value, across all covariates in the model.*

After Benjamini–Hochberg correction the primary associations remained significant — age (P_BH = 0.004) and the CAC score (P_BH = 0.038) for functionality failure, and assisted functionality (P_BH = 0.008) and serum albumin (P_BH = 0.022) for post-functionality AVF failure. Secondary associations were not retained after multiplicity correction (LV diastolic dysfunction P_BH = 0.24 for the primary outcome; diabetes and C-reactive protein P_BH = 0.088 for the secondary outcome) and should be interpreted as hypothesis-generating.

**Supplementary FigureS**

**Figure S1 . Calibration curves for the exploratory predictive model of AVF functionality failure at 90, 120, and 180 days**

**Figure S2 . Decision curve analysis for the exploratory predictive model of AVF functionality failure at 120 days**

**Figure S3 . Calibration curves for the exploratory predictive model of post-functionality AVF failure at 1, 2, and 3 years**

**Figure S4 . Decision curve analysis for the exploratory predictive model of post-functionality AVF failure at 2 years**

**Figure S5 . Collinearity correlation matrix of candidate covariates with the coronary artery calcium score**

**Figure S6 . Adjusted associations of coronary artery calcium score with AVF functionality failure and post-functionality AVF failure under continuous and categorical specifications**

**Figure S7 . Distribution of time from AVF creation to first cannulation**

**Figure S8 . Distribution of coronary artery calcium score by outcome status**

**Figure S9 . Cumulative incidence curves of AVF functionality failure stratified by coronary artery calcium score category**

**Figure S10 . Cumulative incidence curves of post-functionality AVF failure stratified by coronary artery calcium score category**

**Figure S11 . Sensitivity analysis of coronary artery calcium score estimates across model specifications**

**Figure S12 . Cumulative incidence curves comparing primary events and competing events**
